# The phylogenetic distribution of ultraviolet sensitivity in birds

**DOI:** 10.1186/1471-2148-13-36

**Published:** 2013-02-11

**Authors:** Anders Ödeen, Olle Håstad

**Affiliations:** 1 Department of Animal Ecology, Uppsala University, Norbyvägen 18D, Uppsala, S-752 36, Sweden; 2 Department of Anatomy, Physiology and Biochemistry, Swedish University of Agricultural Sciences, P.O. Box 7011, Uppsala, S-750 07, Sweden

## Abstract

**Background:**

Colour vision in birds can be categorized into two classes, the ultraviolet (UVS) and violet sensitive (VS). Their phylogenetic distributions have traditionally been regarded as highly conserved. However, the complicated nature of acquiring spectral sensitivities from cone photoreceptors meant that until recently, only a few species had actually been studied. Whether birds are UVS or VS can nowadays be inferred from a wide range of species via genomic sequencing of the UV/violet SWS1 cone opsin gene.

**Results:**

We present genomic sequencing results of the SWS1 gene from 21 avian orders. Amino acid residues signifying UV sensitivity are found in the two most important spectral tuning sites 86 and 90 of Pteroclidiformes and Coraciiformes, in addition to the major clades, Palaeognathae, Charadriiformes, Trogoniformes, Psittaciformes and Passeriformes, where they where previously known to occur. We confirm that the presumed UVS-conferring amino acid combination F86, C90 and M93 is common to Palaeognathae and unique to this clade, despite available spectrometric evidence showing the ostrich retina to be VS.

**Conclusions:**

By mapping our results together with data from previous studies on a molecular phylogeny we show that avian colour vision shifted between VS and UVS at least 14 times. Single nucleotide substitutions can explain all these shifts. The common ancestor of birds most likely had a VS phenotype. However, the ancestral state of the avian SWS1 opsin’s spectral tuning sites cannot be resolved, since the Palaeognathae are F86, C90 while the Neognathae are ancestrally S86, S90. The phylogenetic distribution of UVS and VS colour vision in birds is so complex that inferences of spectral sensitivities from closely related taxa should be used with caution.

## Background

Vision has played a major role in the evolution of animals. The need to accurately assess the quality of potential mates, spot elusive prey and detect luring predators have driven ecological adaptations in the eyes of vertebrates. The vertebrate eye reflects variation in its environment through the action of pigments in cone and rod photoreceptors with different absorption spectra, which are determined by their respective wavelength of maximum absorption (λ_max_). The visual pigments consist of an opsin protein bound via a Schiff base to a chromophore, either 11-*cis*-retinal or 11-*cis*-3,4-dehydroretinal. Spectral tuning in all but one class of pigment is achieved by replacement of one of the chromophores for the other [1] (11-*cis*-retinal blueshifts compared to 11-*cis*-3,4-dehydroretinal [2,3]), long-pass filtering by pigmented cone oil-droplets [4] or substitutions of key amino acids (aa) in the opsin protein (see refs. in [5]; reviewed by [6]). In the short-wavelength sensitive type 1 pigment (SWS1), λ_max_ is shifted from UVA to violet solely by aa replacements in the pigment opsin, since there is no long-pass filtering by the T-type cone oil droplet in the SWS1 cone [7-9]. Apparently no more is required than a single base substitution, displacing the λ_max_ by 31–47 nm [5,10,11].

In recent years, portable spectrophotometers and the development of vision physiological models have made it possible to quantify how colour signals are perceived by the natural observer, a bird for example, given that visual physiological data for the species in question are known. Since in-depth physiological studies on the visual system have been limited to a few species, researchers have had to rely on a widely accepted assumption of strong phylogenetic inertia in the evolution of colour vision systems, using data from related species, in which the information is available [12].

**Figure 1:**
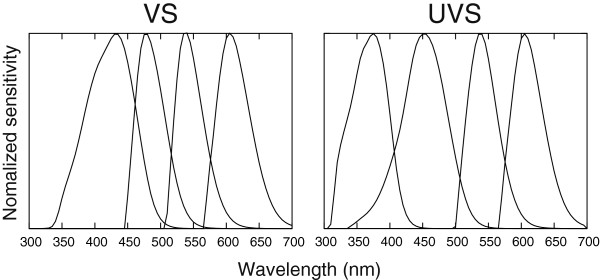
**Examples of spectral sensitivities for VS and UVS birds.** Normalized sensitivities for the single cone classes (from left to right) SWS1, SWS2, MWS and LWS, including the effects of ocular medium absorption. The VS example is the Indian peafowl *Pavo cristatus*[39] and the UVS is the Eurasian blue tit *Cyanistes caeruleus*[9]. Human visible range is approximately 400–700 nm; wavelengths below that range are termed ultraviolet

Diurnal birds, which are highly dependent on colour vision, have evolved two distinct classes of colour vision, the violet sensitive (VS) and the ultraviolet sensitive (UVS) [13]. The foremost difference is that the most shortwave sensitive cone, the SWS1 (UV/violet), has a longwave shifted wavelength of maximum absorption (λ_max_) in the VS class (402–426 nm) compared to the UVS (355–380 nm) (reviewed by [14,15] (Figure 1). The “blue” sensitive SWS2 cones (λ_max_ 451–480 nm) are also longwave shifted in VS birds but to a lesser degree (see review in [14]). Furthermore, the lens and cornea usually show distinctly stronger UV absorption in VS than in UVS species (reviewed in [16,17]). It is important to ecologists to be able to distinguish the two classes of colour vision. Birds use UV cues in both mate choice [18-28] and foraging [29-34]. The UVS class makes the animal more able to discriminate between colours in the natural surroundings (see [35,36]) compared to the VS. For example, parents with UVS vision appear to be better at discriminating colour signals of nestlings [37]. Furthermore, as λ_max_ values differ considerably from those of the VS class, UVS colour vision can grant birds some degree of privacy from VS predators in visual intraspecific communication [38].

Ten years ago it appeared that birds, in contrast to vertebrates as a whole, were ancestrally violet sensitive and that UV sensitivity reappeared once in a common ancestor of passerines and psittaciforms (parrots and allies) [6]. This conclusion was however drawn from less than a dozen bird species that had been investigated through retinal microspectrophotometry (MSP) or *in vitro* regeneration and spectrophotometry of photopigments (see reviews in [14,15]).

Genomic sequencing of small DNA samples has since offered a feasible alternative to gain further insights into the spectral sensitivities of birds. This method is potentially non-destructive and considerably faster to use, compared to MSP and *in vitro* pigment regeneration. The targets are non-conservative substitutions (*i.e.* involving change of charge or loss or gain of a hydroxyl group) that are located in the retinal binding pocket of the opsin protein, on the inner side of its alpha-helices, close enough to directly interact with the Schiff base linkage to the retinal chromophore (e.g. [40]). In the year 2000, two studies, Wilkie et al. [5] and Yokoyama et al. [10], introduced these mutations into the sequence of isolated SWS1 opsin gene from budgerigar *Melopsittacus undulatus* and zebra finch *Taeniopygia guttata*. They showed that replacement of cysteine by serine in the 90^th^ aa position, the substitution C90S, alone accounts for the whole shift in λ_max_ from UVS to VS (all aa numbering in this article corresponds to the bovine rhodopsin [41]). Furthermore, reverse mutations in chicken *Gallus gallus* and pigeon *Columba livia* by Yokoyama et al. [10] produced the full shift from VS to UVS. With these findings at hand we [42] designed primers to PCR amplify fragments from genomic DNA that spanned the proposed major tuning site. From 45 species distributed across 14 bird orders we could confirm in 2003 that ancestral birds likely carried S90 and were hence VS but also that ultraviolet vision had been regained by the S90C substitution not once but four times independently. Ödeen et al. [15] later validated the accuracy of this method in distinguishing UVS from VS species against published MSP data.

The genomic DNA sequences revealed novel combinations of aa residues; although S90 is paired with S86 in all species determined by MSP to be VS, it is combined with A86, I86, C86 or F86 in some other species [42]. These findings lead Carvalho et al. [11] to test alternative substitutions in sites 86 and 90 to UV-shift a pigment *in vitro*, successfully shifting VS pigeon and chicken pigments into the ultraviolet with S86F. Similar to S90C, S86F is a shift from a polar aa residue to a non-polar one in a suitable position to destabilize protonation of the Schiff base linkage between retinal and the opsin, leading to an unprotonated Schiff base, which is typical of UVS pigments [11]. The authors hence implied that the naturally F86, S90 blue-crowned trogon *Trogon curucui* is UVS.

Conferred by the presence of either F86 or C90 aa residues, ultraviolet sensitivity (*i.e.* UVS type opsin genes) may prove to be much more common than previously predicted if the avian SWS1 opsin were sequenced from a denser phylogenetic sample. For this study we have applied genomic sequencing to determine VS or UVS affiliation in species that belong to a number of higher taxa with unknown spectral sensitivities. By compiling the results with published data and mapping these onto a recent phylogeny we trace the evolution of ultraviolet sensitivity in birds.

## Results

**Figure 2:**
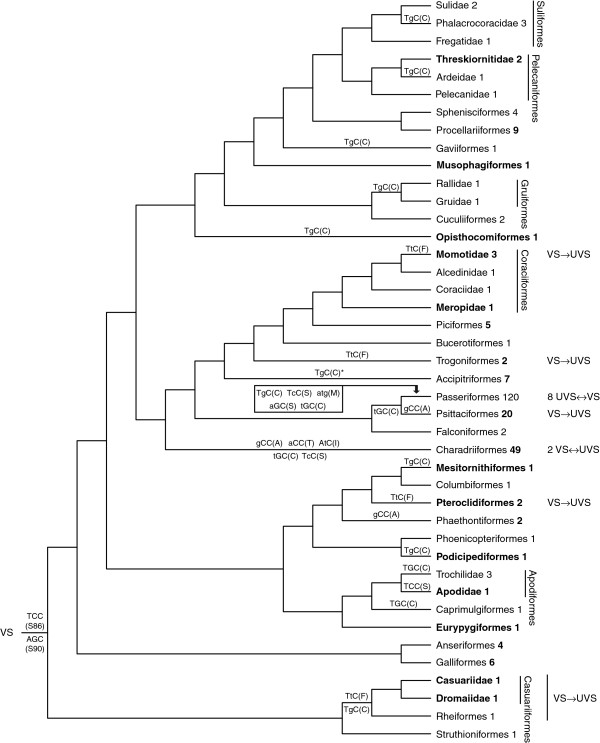
**A phylogenetic reconstruction of SWS1 opsin evolution.** A tree redrawn from Hackett et al. [44], showing shifts between violet (VS) and UV sensitivity (UVS) in SWS1 single-cone pigments (this study and references in text and S2). Taxa new to this study are shown in bold font. In parentheses are the codons and corresponding amino acid residues of the spectral tuning sites 86 (above line) and 90 (below). Nucleotide substitutions (lower case letters) are indicated at their most likely evolutionary position in the tree. The number of species that have been analysed per taxon is shown after taxon names (in bold folt for taxa sequenced in this study). For the sake of brevity, the Charadriiformes and Passeriformes clades have been collapsed. The evolution of SWS1 in these orders is reconstructed in [49] and [50,51], respectively. Asterix (*) indicates that amino acid residue C86 has been found in a subset, family Accipitridae, of the order Accipitriformes.

Cycle sequencing produced 50–160 bp long overlapping sequences of the SWS1 opsin gene from 40 species belonging to 29 families and 21 orders (taxonomy according to the IOC World Bird List [43]), 11 families and six orders being new to this study. We failed to amplify the SWS1 opsin gene in the palaeognath southern brown kiwi *Apteryx australis.* Amino acid translations showing the spectral tuning sites 86, 90 and 93 [5] are presented in Table 1. The phylogenetic distribution of the major tuning aa residues located in sites 86 and 90 are shown in a tree (Figure 2) that is based on the molecular phylogeny of Hackett et al. [44].

**Table 1:** Bird species sequenced for this study

**Order**	**Family**	**Species**	**Common name**	**aa seq 84-94**	**Type**	Origin/voucher*	Tissue no*	**Acc no**
Struthioniformes	Struthionidae	*Struthio camelus*	Common ostrich	FI**F**CVF**C**VF**M**V	VS	Strutsens café	Struts 2012	HF565322
Casuariiformes	Casuariidae	*Casuarius casuarius*	Southern cassowary	FI**F**CVL**C**VF**M**V	US		EBU 46990	HF565323
Casuariiformes	Dromaiidae	*Dromaius novaehollandiae* 2	Emu	FI**F**CVL**C**VF**M**V	VS	O. 70526, O.71207.001	EBU 11410, EBU 45181	HF565324, HF565325
Galliformes	Phasianidae	*Lagopus muta*	Rock ptarmigan	FI**S**CIL**S**VF**V**V	VS	T Sahlman, UU	39	HF565326, HF565327
Galliformes	Phasianidae	*Lagopus lagopus*	Willow ptarmigan	FI**S**CIL**S**VF**V**V	VS	J Höglund, UU	JHGO009	HF565328
Anseriformes	Anatidae	*Branta bernicla*	Brant goose	FI**S**CIF**S**VF**I**V	VS	SVA	682/01	HF565329
Anseriformes	Anatidae	*Cairina moschata*	Muscovy duck	FV**S**CXF**S**VF**I**V	VS	Uppsala kommun	Mysk	HF565330
Anseriformes	Anatidae	*Mergus merganser*	Goosander	FI**S**CIF**S**VF**I**V	VS	IBG, UU	Storskrake	HF565331
Procellariiformes	Procellariidae	*Pterodroma macroptera* 2	Great-winged petrel	FI**S**CIF**S**VF**T**V	VS	–, UWBM 80995	–, CJRR 33466	–^†^, HF565332
Podicipediformes	Podicipedidae	*Podiceps cristatus*	Great crested grebe	FI**C**CIF**S**VF**T**V	VS	SVA	799/01	HF565333
Phaethontiformes	Phaethontidae	*Phaethon rubricauda* 3	Red-tailed tropicbird	FM**A**CIF**S**VF**T**V	VS	–, O. 71305	–, EBU 45522	HM212420^‡^, HM212423^‡^, HF565334
Phaethontiformes	Phaethontidae	*Phaethon lepturus fulvus*	White-tailed tropicbird	FM**A**CIF**S**VF**T**V	VS	O. 71298	EBU 45518	HF565335
Pelecaniformes	Threskiornithidae	*Plegadis falcinellus*	Glossy ibis	FI**S**CIF**S**VF**T**V	VS	NRM 20026066		HF565336
Pelecaniformes	Threskiornithidae	*Platalea ajaja*	Roseate spoonbill	FI**S**CIF**S**VF**T**V	VS	NRM 976748	AHN-354	HF565337
Accipitriformes	Cathartidae	*Cathartes aura ruficollis*	Turkey vulture	FI**S**CIF**S**VF**T**V	VS	NRM 956726		HF565338
Accipitriformes	Accipitridae	*Aviceda subcristata*	Pacific baza	FI**C**CIF**S**VF**I**V	VS	UWBM 76618	EVL 511	HF565339
Accipitriformes	Accipitridae	*Ictinia mississippiensis*	Mississippi kite	FI**C**CIF**S**VF**T**V	VS	UWBM 80091	EVL 706	HF565340
Mesitornithiformes	Mesitornithidae	*Mesitornis unicolor*	Brown mesite	FL**C**CIF**S**VF**T**V	VS	FMNH 346010		HF565341
Eurypygiformes	Rhynochetidae	*Rhynochetos jubatus*	Kagu	FI**S**CVF**S**VF**T**V	VS	O.71868.001		HF565342
Eurypygiformes	Eurypygidae	*Eurypygia helias*	Sunbittern	FI**S**CIF**S**VF**T**V	VS	LSUMZ B38508		HF565343
Charadriiformes	Scolopacidae	*Actitis macularius*	Spotted sandpiper	FI**A**CIF**S**VF**T**V	VS	J Höglund, UU	JHGOx157	AY960714**
Pteroclidiformes	Pteroclididae	*Syrrhaptes paradoxus* 2	Pallas’s sandgrouse	FI**F**CTF**S**VF**T**V	UVS	UWBM 59840, UWBM 59842	CSW 5807	HF565344, HF565345
Pteroclidiformes	Pteroclididae	*Pterocles bicinctus*	Double-banded sandgrouse	FI**F**CSF**S**VF**T**V	UVS	UWBM 53231	SVD 896	HF565346
Columbiformes	Columbidae	*Ptilinopus magnificus*	Wompoo fruit dove	FI**S**CIF**S**VF**T**V	VS	O.73263.001		HF565347
Psittaciformes	Cacatuidae	*Cacatua alba*	White cockatoo	FL**A**CIF**C**IF**T**V	UVS	Djurkliniken Roslagstull		HF565348
Psittaciformes	Cacatuidae	*Nymphicus hollandicus*	Cockatiel	FL**A**CIF**C**IF**T**V	UVS	Fyris Zoo		HF565349
Psittaciformes	Psittacidae	*Aratinga aurea*	Peach-fronted parakeet	FL**A**CIF**C**IF**T**V	UVS	NRM 976646	AHN-308	HF565350
Psittaciformes	Psittacidae	*Pyrrhura frontalis*	Maroon-bellied parakeet	FL**A**CIF**C**IF**T**V	UVS	NRM 966979	GFK-257	HF565351
Psittaciformes	Psittacidae	*Forpus xanthopterygius*	Blue-winged parrotlet	FL**A**CIF**C**IF**T**V	UVS	NRM 986799	LAA-094	HF565352
Opisthocomiformes	Opisthocomidae	*Opisthocomus hoazin*	Hoatzin	FI**C**CIF**S**VF**T**V	VS	LSUMZ B-10753		HF565353
Musophagiformes	Musophagidae	*Tauraco porphyreolophus*	Purple-crested turaco	FI**S**CIF**S**VF**T**V	VS	UWBM 52953	SAR 6784	HF565354
Trogoniformes	Trogonidae	*Harpactes erythrocephalus*	Red-headed trogon	FI**F**CVF**S**VF**T**V	UVS	NRM 20026658	VNM2002-049	HF565355
Coraciiformes	Momotidae	*Momotus momota*	Amazonian motmot	FI**F**CSF**S**VF**T**V	UVS	NRM 947281	ICM-078	HF565356
Coraciiformes	Momotidae	*Baryphthengus ruficapillus*	Rufous-capped motmot	FI**F**CSF**S**VF**T**V	UVS	NRM 937319		HF565357
Coraciiformes	Momotidae	*Eumomota superciliosa*	Turquoise-browed motmot	FI**F**CSF**S**VF**T**V	UVS	NRM 20066359		HF565358
Coraciiformes	Meropidae	*Merops apiaster*	European bee-eater	FV**S**CIF**S**VF**T**V	VS	S Berlin, UU	1/1	HF565359
Piciformes	Megalaimidae	*Megalaima virens*	Great barbet	FI**S**CIF**S**VF**T**V	VS	R den Tex, UU	X-2009	HF565360
Piciformes	Ramphastidae	*Ramphastos tucanus*	White-throated toucan	FI**S**CIF**S**VF**T**V	VS	IBG, UU	Tukan	HF565361
Piciformes	Picidae	*Picus viridis*	European green woodpecker	FL**S**CIF**S**VF**T**V	VS	AÖ	Sko01	HF565362
Passeriformes	Pipridae	*Manacus manacus* 2	White-bearded manakin	FM**C**CIF**S**VF**T**V	VS	L Shorey, Dept Population Biology, UU	LS015, LS241	HF565363, AY227182***

Notes: *Australian Museum (O., EBU), Australian National Wildlife Collection (ANWC), Biology Education Centre, Uppsala university (IBG), Field Museum of Natural History, Chicago (FMNH), Louisiana State University Museum of Natural Science, Baton Rouge (LSUMZ), National Veterinary Institute of Sweden (SVA), Swedish Museum of Natural History (NRM), University of Washington, Burke Museum (UWBM), Uppsala University (UU).

^†^[45], ^‡^[46], **species misidentified in [47], ***re-analysed sample from [42].

The type of SWS1 single cones (VS or UVS) is interpreted from opsin amino acid (aa) identities. The paleognathous Struthioniformes and Casuariformes are assumed to be VS based on MSP data from ostrich *Strutio camelus*[48] and aa sequence similarity. Bold letters mark spectral tuning amino acid positions 86, 90 and 93. Number of birds sequenced is indicated after species names.

We found residues of C90 in the Palaeognathae species ostrich *Strutio camelus,* southern cassowary *Casuarius casuarius* and emu *Dromaius novaehollandiae* (confirming [52]), as well as in the two Cacatuidae and three Psittacidae species, confirming that C90 is common to Psittaciformes (New Zealand parrots, cockatoos and true parrots) (cf. [40,42,52,53]). F86 was found present in the Trogoniformes red-headed trogon *Harpactes erythrocephalus*, all three Momotidae species sampled, rufous-capped motmot *Baryphthengus ruficapillus,* turquoise-browed motmot *Eumomota superciliosa* and blue-crowned motmot *Momotus momota,* and both Pteroclidiformes species sampled, Pallas’s sandgrouse *Syrrhaptes paradoxus* and double-banded sandgrouse *Pterocles bicinctus*, as well as confirmed in the ostrich, southern cassowary and emu (cf. [52], Additional file 1). The combinations A86, C86 or S86 with S90 were common to all the other specimens. Site 93 held threonine in most specimens, isoleucine in some, but methionine in all paleognaths (as in all paleognaths [42,52]).

All Palaeognathae are F86 and C90, coded by TTC and TGC, while serine in both site 86 and the 90, coded by TCC and AGC respectively, is the most parsimonious ancestral state for the Neognathae (the remaining taxa) (Figure 2). Single nucleotide substitutions from AGC to TGC appear to have occurred in the ancestors of C90 species and other single nucleotide substitutions have resulted in the aa substitutions S86F, S86C and S86A. All aa changes in sites 86 and 90 may be parsimoniously interpreted as non-conservative substitutions.

Our rock ptarmigan *Lagopus muta* sequences differ by three out of eleven aa residues from that published in Håstad et al. [47] (Table 1). Their sequence originated from the same sample as our spotted sandpiper *Actitis macularius* but was misidentified due to a case of mislabelling confirmed by the collection manager. The ostrich and white-bearded manakin *Manacus manacus* aa sequences reported here are similar to those recently published by Aidala et al. [52] but not compared to an earlier study (Ödeen and Håstad [42]). Through re-sequencing and mtDNA barcoding of the samples from [42] we have determined that the ostrich sample was misidentified at the source and the manakin during sequencing. Two aa residues in the manakin sequence in [42] are hereby corrected but the change (S86 to C86) is not likely relevant to spectral tuning (cf. [11]). The revision of the ostrich however changes what was believed to be a VS S86, S90 coding genotype into a supposedly UVS F86, C90, common to other paleognaths.

## Discussion

With the results of this study, information has become available on the SWS1 opsin’s VS-UVS state for most higher order avian taxa.

Avian evolution has seen at least 14 shifts between VS and UVS colour vision (this study and [5,15,16,40,42,45-47,49-60]). If Passeriformes and Psittaciformes are sister orders (Figure 2[44]), UV vision has been regained eight or nine times. Serine in site 90 has been substituted by cysteine (S90C) five or six times, depending on the number of reverse substitutions (see below): in a charadriiform ancestor of *Larus* gulls and allies gulls, *Anous* and *Gygis* terns, and black skimmer *Rynchops niger*[49], in an ancestor of Psittaciformes (see [40,42,52,53]) and Passeriformes, and three or four times in Passeriformes [50,51]. Ultraviolet shifts by the substitution S86F, without S90C, seem to have taken place three times (Figure 2): in Pteroclidiformes, Trogoniformes (see also [42]) and Coraciiformes (Momotidae). It should be noted however that the *in vitro* shift resulting from S86F in chicken and pigeon pigments [11] remains to be confirmed *in vivo* by e.g. MSP in such a taxon where F86 occurs naturally. There is evidence of five or six reversals to VS by C90S in Aves, depending on the number of S90C: one in the Charadriiformes [49] and four (if four S90C) or five (if three S90C) in Passeriformes [50,51].

The ancestral aa residues cannot be reconstructed for the two most important spectral tuning sites of the avian SWS1 opsin. This study supports recent findings by Aidala et al. [52] that all paleognaths hold F86 and C90 but suggests by parsimony (Figure 2) that S86 plus S90 was present in the common ancestor of all other extant birds (Neognathae). However, the common ancestor of birds was most likely VS rather than UVS. The only paleognathous species that has been investigated by MSP, the ostrich, has VS SWS1 single cones [48]. By priority of direct evidence (MSP) over indirect (genomic sequencing) we conclude that Palaeognathae have VS photopigments, and thus that one or more unknown aa’s in the SWS1 opsin stabilizes protonation of the Schiff base linkage to the retinal despite the presence of C90. A likely candidate is the nonpolar methionine that paleognaths share at site 93 (Additional file 1). Another nonpolar aa at site 93, proline, has recently been shown to be important to primate VS pigments [59]; the substitution P93T shifts the VS aye-aye *Daubentonia madagascariensis* pigment’s λ_max_ into the UV. In addition to Palaeognathae, Laridae and *Acanthisitta chloris* have C90 together with nonpolar aa residues (I or L and L, respectively) at site 93, and might therefore have VS phenotypes due to similar stabilizing effects on Schiff base protonation. However, it is known from site-directed mutagenesis [5] that the polar to nonpolar substitution T93V only has a very minor effect (3 nm) on spectral tuning of a C90 SWS1 opsin pigment. We are therefore reluctant to assume a general, strong effect of nonpolar aa’s at site 93 in birds until it is supported by *in vitro* expression or MSP.

Point mutations can explain almost all aa substitutions at sites 86 and 90. The S90C induced UV shifts seem to have been caused by the codon AGC changing to TGC, while both AGC and TCC have been responsible for C90S reversals to violet (see [49-51]. Other single nucleotide substitutions have resulted in S86F, S86C and S86A. A parsimonious interpretation, consistent with the phylogenetic reconstruction of ultraviolet vision in Charadriiformes (shorebirds) by Ödeen et al. [49], is that point mutations also caused A86T in the ancestor of *Sterna* and allies terns, and then T86I in the ancestor of gulls. The only exception seems to be a triple nucleotide substitution from TCC to ATG (C86M) (see [51]) in a common ancestor of the Passerida, Sylvoidea passerine families (*sensu* Johansson et al. [61]) Pycnonotidae, Hirundidae, Phylloscopidae, Acrocephalidae, Donacobidae, Timaliidae and Zosteropidae.

Despite 14 shifts between violet and UV vision during avian evolution, the simple nature of the mechanism behind them makes one wonder why some clades are invariably VS or UVS; all that is required for a shift is apparently a point mutation [5,10,11]. With the exception of *Malurus* fairywrens [50], multi-sampled avian genera show no polymorphism in SWS1 λ_max_, but hold either VS or UVS type sequences (Additional file 1).

VS–UVS shifts in the SWS1 pigment may have to happen in correlation with other changes in the physiology of the eye to be positively selected. A shortwave shift in a VS SWS1 pigment should not produce a significant increase in UV sensitivity unless it is preceded by an increased transmission of UV in the ocular media (a lowered cut-off wavelength (λ_T0.5_) [16]. There may be a cost associated with increased UV transmission as shortwave radiation is absorbed by and photooxidizes biological tissue (e.g. [53,62]). The ocular media of most VS birds filter out some of the ultraviolet radiation, thereby limiting the photooxidative damage on the retina. Reduced visual acuity and contrast by chromatic aberration and Rayleigh scattering takes an additional toll on widening spectral transmission to shorter wavelengths [63]. Moreover, single-cone sensitivities would become unevenly distributed across the spectrum, and thus to colour discriminability suboptimally positioned [35], without a compensatory shortwave shift in the SWS2 pigment. The latter should prove a relatively gradual process, requiring the additive effect of at least five spectral tuning aa substitutions in its opsin [64] compared to a single nucleotide substitution in the SWS1 [5,10,11]. Conversely, a prerequisite of compensatory longwave shifts in SWS2 may explain why the SWS1 in some clades is invariably UVS, *i.e.* in Charadriiformes [49], in Passeriformes [51] and Psittaciformes (e.g. [52,53]).

Multifocal optics may form another constraint to substantial but isolated shifts in single cone λ_max_. Examining 45 species from 12 orders, Lind et al. [65] has demonstrated that birds, with few exceptions, have multifocal lenses. These consist of concentric zones with different refractive powers, which serve to reduce longitudinal chromatic aberration by selectively focusing multiple narrow wavelength bands onto the retina. As in the African cichlid *Astatotilapia burtoni*, where multifocal lenses were first described, these bands may closely correspond to the respective λ_max_ of the single cone classes present in the retina [66]. An isolated and sizeable shift in λ_max_ of any single cone class that is not optically corrected for should in such an eye produce a mismatch with the refractive index of the dedicated zone in the lens. The result would be chromatic blur and deteriorated spatial resolution due to an inability to simultaneously focus white light onto all single cone classes. Because longitudinal chromatic aberration increases towards the shortwave end of the spectrum (see [66]) this effect would apply to the SWS1, ultraviolet/violet sensitive, cone class in particular.

## Conclusions

It is clear that avian colour vision systems are not as conserved as previously believed. To infer spectral sensitivity of a bird from a closely related species, in which the information is available, is probably still a tenable approach in large, comparative, cross-species studies. However, when the focus is one or a few particular species, it is advisable to acquire at least the most variable vision physiological data, *i.e.* VS or UVS colour vision system affiliation. It is particularly important when the study species are closely related to clades of taxa with identified shifts in the colour vision system, such as Laridae charadriiforms [49] and non-Passerida/non-Petroicidae passerines [50,51].

As ultraviolet and violet vision are important to mate choice, foraging and predator avoidance [18-34], spectral tuning of the SWS1 cone opsin presents a very rare insight into the molecular processes of ecological adaptation. We may get a clearer view of the evolution of colour vision with additional data on SWS2 sensitivities and on ocular transmission, as well as refractive indices in compound lenses. That could help to explain how shortwave colour vision has evolved, determining whether major shifts in the spectral sensitivity of the SWS1 pigment are facilitated and/or constrained by shifts in co-adapted physiological traits. Beyond the realms of eye physiology and molecular biology, deeper investigations in ecology (such as [37,38,47,67] are needed to understand its role in shifting VS to UVS.

## Methods

We extracted genomic DNA from quill bases of feathers, blood, muscle and other tissue material either with a GeneMole® automated nucleic acid extraction instrument (Mole Genetics), the DNeasy Blood and Tissue Kit (QIAGEN) or with Chelex. Standard procedures were applied except for the quill bases, which were lysated with 1% DTT. Feather material was sampled from a European green woodpecker *Picus viridis* killed by traffic. Live animals were not sampled for this study. Other tissue material was borrowed from museum collections and from the collections of colleagues, the National Veterinary Institute SVA in Uppsala and Uppsala City Council. We performed mtDNA barcoding with COI, following the Stockholm protocol outlined in [68], to confirm labelling of selected tissue samples and to identify species *Ramphastos tucanus* from an unspecified toucan tissue sample.

Using the degenerate primers SU80a [69], SU149a, SU161a, SU193a [42] or SU200Ca, combined with SU304b [15] or SU306b [42] we amplified a gene fragment coding for residues from aa sites 81–94, located in the 2nd α-helical transmembrane region of the SWS1 opsin. We conducted PCR on an Eppendorf MasterCycler Gradient or a PE Applied Biosystems Geneamp® PCR System 9700 with reactions containing 0.5-2.5 ng/μl DNA extracts, 1 unit Taq-polymerase (Applied Biosystems) plus reaction buffer, 0.4 pmol of forward and reverse primers, 0.2 mM of each dNTP, and 2 mM MgCl_2_. Each PCR reaction contained 0.5–2.5 *n*g/*μ*l total DNA extracts, 1 unit Taq-polymerase (Applied Biosystems) with reaction buffer, 0.4 *p*mol of forward and reverse primers, 0.2 *m*M of each dNTP, and 2 *m*M MgCl_2_. For some reactions, PuReTaq™ Ready-To-Go™ PCR beads (GE Healthcare) replaced separate volumes of Taq-polymerase, dNTP’s and MgCl_2_. Initially, the reaction conditions followed [42] (*i.e.* 90 s at 94°C, 5 × (30 s at 94°C, 30 s at 54°C and 1 s at 72°C), 38 × (15 s at 94°C, 30 s at 54°C and 5 s at 72°C) and 10 min at 72°C) but were later optimized to exclude the extension phase in order to minimize nonspecific amplification of longer fragments. The final version of thermocycling started with 90 s at 94°C, was followed by 48 × (5 s at 94°C, 15 s at 54°C) and ended with 1 s at 72°C. We used a different protocol for the primer pair SU80a/SU306b, namely 2 min 30 s at 95°C, 40 × (30 s at 95°C, 30 s at 54°C and 10 s at 72°C) and 1 min at 72°C. Two percent agarose gel electrophoresis for 90 min at 80 V confirmed amplification and expected fragment length. When there were extra fragments present we sometimes performed a second PCR on the products using internal primers.

The PCR products were purified with EXOsap-IT (USB). Macrogen Inc. (South Korea) then performed double-stranded sequencing using the same primers as above plus SU200a [15], SU200Ga [60], and SU296b 5^′^-AAG AYR AAG TAD CCS YGS G-3^′^, which we designed for this study with the help of Primer3 online software (http://frodo.wi.mit.edu/) [70].

We translated our DNA sequences into aa’s to identify the spectral tuning sites 86, 90, and 93 [5,10]. From the aa residues presents at these sites we estimated λ_max_ values following Wilkie et al. [5], Yokoyama et al. [10] and Carvalho et al. [11] as outlined in [15].

## Abbreviations

λmax: Wavelength of maximum absorption; SWS1: Short wavelength sensitive type one; SWS2: Short wavelength sensitive type 2; MWS: Medium wavelength sensitive; LWS: Long-wavelength sensitive; UVS: Ultraviolet sensitive; VS: Violet sensitive; MSP: Microspectrophotometry.

## Competing interest

The authors declare that they have no competing interests.

## Authors’ contributions

AÖ and OH conceived of the study. AÖ compiled the tissue material, carried out the molecular analyses, interpreted the opsin results and drafted the manuscript together with OH. Both authors finalised and approved the manuscript.

## Supplementary Material

Additional file 1 **Table with type of SWS1 single cones (VS or UVS) interpreted from avian opsin amino acid (aa) sequences.** Bold letters mark spectral tuning amino acid positions 86, 90 and 93. Amino acid residues without a known spectral tuning effect are noted. Number of birds sequenced is indicated after species names. *See note below table. Click here for file
