# Correction: The phylogenetic distribution of ultraviolet sensitivity in birds

**DOI:** 10.1186/1471-2148-14-62

**Published:** 2014-03-28

**Authors:** Anders Ödeen, Olle Håstad

**Affiliations:** 1 Department of Animal Ecology, Uppsala University, Norbyvägen 18D, Uppsala S-752 36, Sweden; 2 Department of Anatomy, Physiology and Biochemistry, Swedish University of Agricultural Sciences, P.O. Box 7011, Uppsala S-750 07, Sweden

## 

After publication of this work, it has come to our attention that inaccuracies are present in the HTML version of Figure two (Figure 1 here) in the published manuscript. The correct version of this figure is included in this erratum. Note that the PDF version of the original publication contains the correct version of Figure two (Figure 1 here).

**Figure 1:**
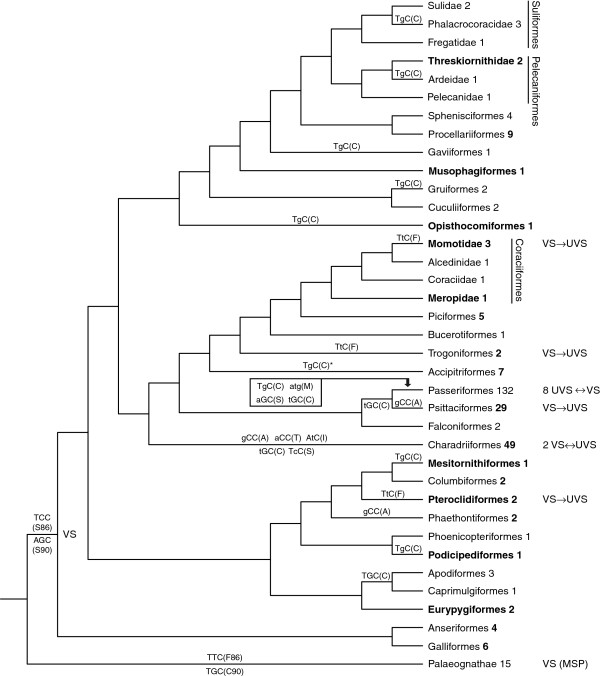
**Correct version of Figure two (Figure**1**here) from Ödeen and Håstad 2013 **[1]**.** Reference numbers in the following legend refer to the original article. A phylogenetic reconstruction of SWS1 opsin evolution. A tree redrawn from Hackett et al. [44], showing shifts between violet (VS) and UV sensitivity (UVS) in SWS1 single-cone pigments (this study and references in text and S2). Taxa new to this study are shown in bold font. In parentheses are the codons and corresponding amino acid residues of the spectral tuning sites 86 (above line) and 90 (below). Nucleotide substitutions (lower case letters) are indicated at their most likely evolutionary position in the tree. The number of species that have been analysed per taxon is shown after taxon names (in bold folt for taxa sequenced in this study). For the sake of brevity, the Charadriiformes and Passeriformes clades have been collapsed. The evolution of SWS1 in these orders is reconstructed in [49] and [50,51], respectively. Asterix (*) indicates that amino acid residue C86 has been found in a subset, family Accipitridae, of the order Accipitriformes.

These errors were introduced during the final production process and the editors of *BMC Evolutionary Biology* apologise for any inconvenience caused.
