# Supplementary material for: The phylogenetic distribution of ultraviolet sensitivity in birds
Source: BMC Evol Biol. 2013 Feb 11;13:36. doi: 10.1186/1471-2148-13-36 (PMC3637589; doi:10.1186/1471-2148-13-36)
Supplement: Additional file 1 — Table with type of SWS1 single cones (VS or UVS) interpreted from avian opsin amino acid (aa) sequences. Bold letters mark spectral tuning amino acid positions 86, 90 and 93. Amino acid residues without a known spectral tuning effect are noted. Number of birds sequenced is indicated after species names. *See note below table. [file 1471-2148-13-36-S1.doc]

# Additional file 1

Table with type of SWS1 single cones (VS or UVS) interpreted from avian opsin amino acid (aa) sequences. Bold letters mark spectral tuning amino acid positions 86, 90 and 93. Amino acid residues without a known spectral tuning effect are noted. Number of birds sequenced is indicated after species names. *, **See notes below table.

| **Order** | **Family** | **Species** | **Common name** | **aa seq 84-94** | **Type** | **Unkn effect aa** | **Origin/voucher no*** | **Tissue no*** | **Acc no** | **Reference** |
| --- | --- | --- | --- | --- | --- | --- | --- | --- | --- | --- |
| Dinornithiformes | Emeidae | *Emeus crassus* | Eastern moa | FI**F**CIF**C**VF**X**X | VS** |  |  |  |  | [1] |
| Dinornithiformes | Emeidae | *Euryapteryx curtus* | Stout-legged moa | FI**F**CIF**C**VF**X**X | VS** |  |  |  |  | [1] |
| Dinornithiformes | Emeidae | *Euryapteryx gravis* | NI broad-billed moa | FI**F**CIF**C**VF**X**X | VS** |  |  |  |  | [1] |
| Dinornithiformes | Emeidae | *Pachyornis elephantopus* | Heavy-footed moa | FI**F**CIF**C**VF**X**X | VS** |  |  |  |  | [1] |
| Dinornithiformes | Emeidae | *Pachyornis geranoides* | Mantells moa | FI**F**CIF**C**VF**X**X | VS** |  |  |  |  | [1] |
| Tinamiformes | Tinamidae | *Tinamus major* | Great tinamou | FI**F**CCF**C**VF**M**X | VS** | M93 |  |  |  | [1] |
| Tinamiformes | Tinamidae | *Crypturellus undulatus* | Undulated tinamou | FV**F**CVF**C**VF**M**X | VS** | M93 |  |  |  | [1] |
| Tinamiformes | Tinamidae | *Rhynchotus rufescens* | Red-winged tinamou | FI**F**CVF**C**VF**M**X | VS** | M93 |  |  |  | [1] |
| Tinamiformes | Tinamidae | *Nothoprocta ornata* | Ornate tinamou | FI**F**CVF**C**VF**M**X | VS** | M93 |  |  |  | [1] |
| Tinamiformes | Tinamidae | *Nothoprocta pentlandii* | Andean tinamou | FI**F**CCF**C**VF**M**X | VS** | M93 |  |  |  | [1] |
| Tinamiformes | Tinamidae | *Nothura boraquira* | White-bellied nothura | FI**F**CVF**C**VF**M**X | VS** | M93 |  |  |  | [1] |
| Struthioniformes | Struthionidae | *Struthio camelus* | Common ostrich | FI**F**CVF**C**VF**M**X | VS** | M93 |  |  |  | [1] |
| Struthioniformes | Struthionidae | *Struthio camelus* | Common ostrich | FI**F**CVF**C**VF**M**V | VS** | M93 | Strutsens café | Struts 2012 | HF565322 | This study |
| Rheiformes | Rheidae | *Rhea americana* | Greater rhea | FI**F**CFF**C**VF**M**V | VS** | M93 | IBG, UU |  | AY227188 | [2] |
| Rheiformes | Rheidae | *Rhea americana* | Greater rhea | FI**F**CFF**C**VF**X**X | VS** | M93 |  |  |  | [1] |
| Casuariiformes | Casuariidae | *Casuarius casuarius* | Southern cassowary | FI**F**CVL**C**VF**M**X | VS** | M93 |  |  |  | [1] |
| Casuariiformes | Casuariidae | *Casuarius casuarius* | Southern cassowary | FI**F**CVL**C**VF**X**X | VS** | M93 |  |  |  | [1] |
| Casuariiformes | Casuariidae | *Casuarius casuarius* | Southern cassowary | FI**F**CVL**C**VF**M**V | VS** | M93 |  | EBU 46990 | HF565323 | This study |
| Casuariiformes | Dromaiidae | *Dromaius novaehollandiae* | Emu | FI**F**CVL**C**VF**M**V | VS** | M93 |  |  |  | [1] |
| Casuariiformes | Dromaiidae | *Dromaius novaehollandiae* 2 | Emu | FI**F**CVL**C**VF**M**V | VS** | M93 | O.70526, O.71207.001 | EBU 11410, EBU 45181 | HF565324, HF565325 | This study |
| Galliformes | Phasianidae | *Meleagris gallopavo* | Wild turkey | FV**S**CVL**S**VF**V**V | VS |  | N Backström, S Berlin | T9 | FJ440635 | [3] |
| Galliformes | Phasianidae | *Lagopus muta* 2 | Rock ptarmigan | FI**S**CIL**S**VF**V**V | VS |  | T Sahlman, Dept Population Biology, UU | 1, 39 | HF565326, HF565327 | This study |
| Galliformes | Phasianidae | *Lagopus lagopus* | Willow ptarmigan | FI**S**CIL**S**VF**V**V | VS |  | J Höglund, Dept Population Biology, UU | JHGO009 | HF565328 | This study |
| Galliformes | Phasianidae | *Coturnix japonica* | Japanese quail | FV**S**CVL**S**VF**V**V | VS |  |  |  | AY227172 | [2] |
| Galliformes | Phasianidae | *Gallus gallus* | Domestic chicken | FV**S**CVL**S**VF**V**V | VS |  |  |  | M92039 | [4] |
| Galliformes | Phasianidae | *Pavo cristatus* | Indian peafowl | FV**S**CVL**S**VF**V**V | VS |  | Borås Zoo |  | FJ440636 | [3] |
| Anseriformes | Anatidae | *Branta bernicla* | Brant goose | FI**S**CIF**S**VF**I**V | VS | I93 | SVA | 682/01 | HF565329 | This study |
| Anseriformes | Anatidae | *Cairina moschata* | Muscovy duck | FV**S**CXF**S**VF**I**V | VS | I93 | Uppsala kommun/Dept Animal Ecology, UU | Mysk | HF565330 | This study |
| Anseriformes | Anatidae | *Anas platyrhynchos* | Mallard | FV**S**CIF**S**VF**I**V | VS | I93 | Lindeberg & von Schantz, Uppsala |  | AY227147 | [3] |
| Anseriformes | Anatidae | *Mergus merganser* | Goosander | FI**S**CIF**S**VF**I**V | VS | I93 | IBG, UU | Storskrake | HF565331 | This study |
| Sphenisciformes | Spheniscidae | *Aptenodytes patagonicus* | King penguin | FI**S**CIF**S**VF**I**V | VS | I93 |  |  |  | [5] |
| Sphenisciformes | Spheniscidae | *Pygoscelis adeliae* | Adelie penguin | FV**S**CIF**S**VF**T**V | VS |  | A Bartosch-Härlid, Dept Evolutionary Biology, UU |  | AY227167 | [2] |
| Sphenisciformes | Spheniscidae | *Eudyptula minor* | Little penguin | FI**S**CIF**S**VF**T**V | VS |  |  |  |  | [5] |
| Sphenisciformes | Spheniscidae | *Spheniscus humboldti* | Humboldt penguin | FI**S**CIF**S**VF**T**V | VS |  |  |  | AJ277991 | [6] |
| Gaviiformes | Gaviidae | *Gavia stellata* | Red-throated loon | FI**C**CIF**S**VF**T**V | VS |  | SVA | 81/01 | AY227158 | [2] |
| Procellariiformes | Diomedeidae | *Diomedea exulans* | Wandering albatross | FI**S**CIF**S**VF**T**V | VS |  |  |  |  | [5] |
| Procellariiformes | Diomedeidae | *Diomedea epomophora* | Southern royal albatross | FI**S**CIF**S**VF**T**V | VS |  |  |  |  | [5] |
| Procellariiformes | Diomedeidae | *Thalassarche chlororhynchos* | Atlantic yellow-nosed albatross | FI**S**CIF**S**VF**T**V | VS |  |  |  |  | [5] |
| Procellariiformes | Procellariidae | *Fulmarus glacialis* | Northern fulmar | FI**S**CIF**S**VF**T**V | VS |  |  |  | AY960715 | [7] |
| Procellariiformes | Procellariidae | *Pterodroma macroptera* 2 | Great-winged petrel | FI**S**CIF**S**VF**T**V | VS |  | –, UWBM 80995 | –, CJRR 33466 | –, HF565332 | [5], this study |
| Procellariiformes | Procellariidae | *Puffinus pacificus* | Wedge-tailed shearwater | FI**S**CIF**S**VF**T**V | VS |  | O.71274 | EBU 45592 | FJ440633 | [3] |
| Procellariiformes | Procellariidae | *Puffinus puffinus* | Manx shearwater | FI**S**CIF**S**VF**T**V | VS |  | NRM 20056441 |  | FJ440634 | [3] |
| Procellariiformes | Hydrobatidae | *Pelagodroma marina* | White-faced storm petrel | FI**S**CIF**S**VF**T**V | VS |  |  |  |  | [5] |
| Procellariiformes | Hydrobatidae | *Oceanodroma leucorhoa* | Leach's storm petrel | FI**S**CIF**S**VF**T**V | VS |  | A Bartosch-Härlid, Dept Evolutionary Biology, UU |  | AY227166 | [2] |
| Podicipediformes | Podicipedidae | *Podiceps cristatus* | Great crested grebe | FI**C**CIF**S**VF**T**V | VS |  | SVA | 799/01 | HF565333 | This study |
| Phoenicopteriformes | Phoenicopteridae | *Phoenicopterus ruber* | American flamingo | FV**S**CVL**S**VF**V**V | VS |  | IBG, UU |  | AY227165 | [2] |
| Phaethontiformes | Phaethontidae | *Phaethon rubricauda* 3 | Red-tailed tropicbird | FM**A**CIF**S**VF**T**V | VS |  | –, O. 71305 | –, EBU 45522 | HM212420, HM212423, HF565334 | [8], this study |
| Phaethontiformes | Phaethontidae | *Phaethon lepturus fulvus* | White-tailed tropicbird | FM**A**CIF**S**VF**T**V | VS |  | O.71298 | EBU 45518 | HF565335 | This study |
| Pelecaniformes | Threskiornithidae | *Plegadis falcinellus* | Glossy ibis | FI**S**CIF**S**VF**T**V | VS |  | NRM 20026066 |  | HF565336 | This study |
| Pelecaniformes | Threskiornithidae | *Platalea ajaja* | Roseate spoonbill | FI**S**CIF**S**VF**T**V | VS |  | NRM 976748 | AHN-354 | HF565337 | This study |
| Pelecaniformes | Ardeidae | *Ardea cinerea* | Grey heron | FI**C**CIF**S**VF**T**V | VS |  | SVA | 420/01 | AY227153 | [2] |
| Pelecaniformes | Pelecanidae | *Pelecanus onocrotalus* | Great white pelican | FX**S**CXF**S**VF**T**V | VS |  | SVA |  | AY960713 | [7] |
| Suliformes | Fregatidae | *Fregata minor* 2 | Great frigatebird | FI**S**CIF**S**VF**T**V | VS |  |  |  | EU651855, EU651856 | [9] |
| Suliformes | Sulidae | *Morus bassanus* | Northern gannet | FI**S**CIF**S**VF**T**V | VS |  |  |  | AY960721 | [7] |
| Suliformes | Sulidae | *Morus serrator* | Australian gannet | FI**S**CIF**S**VF**T**V | VS |  |  |  |  | [5] |
| Suliformes | Phalacrocoracidae | *Microcarbo melanoleucos* | Little pied cormorant | FI**C**CLF**S**VF**T**V | VS |  |  |  |  | [5] |
| Suliformes | Phalacrocoracidae | *Phalacrocorax sulcirostris* | Little black cormorant | FI**C**CLF**S**VF**T**V | VS |  |  |  |  | [5] |
| Suliformes | Phalacrocoracidae | *Phalacrocorax carbo* | Great cormorant | FI**C**CLF**S**VF**T**V | VS |  |  |  | AY227164, – | [2,5] |
| Accipitriformes | Cathartidae | *Cathartes aura ruficollis* | Turkey vulture | FI**S**CIF**S**VF**T**V | VS |  | NRM |  | HF565338 | This study |
| Accipitriformes | Pandionidae | *Pandion haliaetus* | Western osprey | FI**S**CIF**S**VF**T**V | VS |  | SVA | 1213/01 | AY227152 | [2] |
| Accipitriformes | Accipitridae | *Aviceda subcristata* | Pacific baza | FI**C**CIF**S**VF**I**V | VS | I93 | UWBM 76618 | EVL 511 | HF565339 | This study |
| Accipitriformes | Accipitridae | *Ictinia mississippiensis* | Mississippi kite | FI**C**CIF**S**VF**T**V | VS |  | UWBM 80091 | EVL 706 | HF565340 | This study |
| Accipitriformes | Accipitridae | *Circus aeruginosus* | Western marsch harrier | FI**S**CIF**S**VF**T**V | VS |  | SVA | 673/01 | AY227151 | [2] |
| Accipitriformes | Accipitridae | *Accipiter nisus* | Eurasian sparrowhawk | FI**S**CIF**S**VF**T**V | VS |  | S Berlin, Dept Evolutionary Biology, UU |  | AY227149 | [2] |
| Accipitriformes | Accipitridae | *Accipiter gentilis* | Northern goshawk | FI**X**CIF**S**VF**T**V | VS |  | SVA | 1212/01 | AY227148 | [2] |
| Accipitriformes | Accipitridae | *Buteo buteo* | Common buzzard | FI**S**CIF**S**VF**T**V | VS |  | SVA | 866/01 | AY227150 | [2] |
| Falconiformes | Falconidae | *Caracara cheriway* | Southern crested caracara | FI**S**YTF**S**VF**T**V | VS |  | FMNH 393524 |  | HE601811 | [10] |
| Falconiformes | Falconidae | *Falco peregrinus* | Peregrine falcon | FI**S**CIF**S**VF**T**V | VS |  |  | NRM A1995.6079 | AY227157 | [2] |
| Mesitornithiformes | Mesitornithidae | *Mesitornis unicolor* | Brown mesite | FL**C**CIF**S**VF**T**V | VS |  | FMNH 345610 |  | HF565341 | This study |
| Eurypygiformes | Rhynochetidae | *Rhynochetos jubatus* | Kagu | FI**S**CVF**S**VF**T**V | VS |  | O.71868.001 |  | HF565342 | This study |
| Eurypygiformes | Eurypygidae | *Eurypyga helias* | Sunbittern | FI**S**CIF**S**VF**T**V | VS |  | LSUMZ B38508 |  | HF565343 | This study |
| Gruiformes | Rallidae | *Fulica atra* | Eurasian coot | FL**C**CIF**S**VF**T**V | VS |  | SVA | 458/01 | AY227175 | [2] |
| Gruiformes | Gruidae | *Balearica pavonina* | Black crowned crane | FI**C**CIF**S**VF**T**V | VS |  | IBG, UU |  | AY227174 | [2] |
| Charadriiformes | Haematopodidae | *Haematopus ostralegus* | Eurasian oystercatcher | FI**A**CIF**S**VF**T**V | VS |  |  |  | AY227155 | [2] |
| Charadriiformes | Recurvirostridae | *Himantopus himantopus* | Black-winged stilt | FV**A**CIF**S**VF**T**V | VS |  | A Lindström & AÖ | It09 | AY227156 | [2] |
| Charadriiformes | Charadriidae | *Pluvialis apricaria* | Eurasian golden plover | FI**A**CIF**S**VF**T**V | VS |  | Dept Animal Ecology, UU | Ljungpipare98 | GU129689 | [11] |
| Charadriiformes | Charadriidae | *Charadrius dubius* | Little ringed plover | FI**A**CIF**S**VF**T**V | VS |  | A Lindström & AÖ | Ma01 | AY227154 | [2] |
| Charadriiformes | Thinocoridae | *Thinocorus orbignyianus* | Grey-breasted Seedsnipe | FI**A**CIF**S**VF**T**V | VS |  | UWBM 54407 | DAB 808 | GU129666 | [11] |
| Charadriiformes | Scolopacidae | *Gallinago gallinago* | Common snipe | FI**A**CIF**S**VF**T**V | VS |  |  |  | AY960717 | [7] |
| Charadriiformes | Scolopacidae | *Tringa glareola* | Wood sandpiper | FI**A**CIF**S**VF**T**V | VS |  | Dept Animal Ecology, UU | 031218-2 | GU129664 | [11] |
| Charadriiformes | Scolopacidae | *Actitis hypoleucos* | Common sandpiper | FI**A**CIF**S**VF**T**V | VS |  | J Höglund, Dept Population Biology, UU | JHGO107 | AY960716 | [7] |
| Charadriiformes | Scolopacidae | *Actitis macularius* | Spotted sandpiper | FI**A**CIF**S**VF**T**V | VS |  | J Höglund, Dept Population Biology, UU | JHGOx157 | AY960714 | This study; misidentified in [7] |
| Charadriiformes | Scolopacidae | *Philomachus pugnax* 11 | Ruff | FI**A**CIF**S**VF**T**V | VS |  | F Widemo | 11 individuals | GU129665 | [11] |
| Charadriiformes | Scolopacidae | *Phalaropus fulicarius* | Red phalarope | FI**A**CIF**S**VF**T**V | VS |  | Dept Population Biology, UU | JHGO30 | AY960718 | [7] |
| Charadriiformes | Glareolidae | *Rhinoptilus chalcopterus* | Bronze-winged Courser | CL**A**CLF**S**VF**T**V | VS |  | ZMUC 131583 |  | GU129688 | [11] |
| Charadriiformes | Glareolidae | *Glareola pratincola* | Collared pratincole | FL**A**CVF**S**VF**T**V | VS |  | M Wilson & AÖ | Ug35 | GU129686 | [11] |
| Charadriiformes | Glareolidae | *Glareola nuchalis* | Rock Pratincole | FL**A**CIF**S**VF**T**V | VS |  | ZMUC 113386 |  | GU129687 | [11] |
| Charadriiformes | Laridae | *Anous tenuirostris* | Lesser noddy | FI**A**CIF**C**IF**T**V | UVS |  | ZMUC 113339 |  | GU129675 | [11] |
| Charadriiformes | Laridae | *Anous minutus* | Black noddy | FI**A**CIF**C**IF**T**V | UVS |  | ZMUC 137802 |  | GU129674 | [11] |
| Charadriiformes | Laridae | *Rynchops niger* | Black skimmer | FV**A**CVF**C**IF**T**V | UVS |  | LSUMZ B-2457 |  | GU129673 | [11] |
| Charadriiformes | Laridae | *Gygis alba* | White tern | FI**A**CIF**C**IF**T**V | UVS |  | ZMUC 113340 |  | GU129678 | [11] |
| Charadriiformes | Laridae | *Creagrus furcatus* | Shallow-tailed gull | FI**I**CVF**C**IS**I**V | UVS | I86, 93 | LSUMZ B-15450 |  | GU129672 | [11] |
| Charadriiformes | Laridae | *Rissa tridactyla* | Black-legged kittiwake | FI**I**CVF**C**IS**I**V | UVS | I86, 93 |  |  | AY960712 | [7] |
| Charadriiformes | Laridae | *Pagophila eburnea* | Ivory gull | FI**I**CVF**C**IS**I**V | UVS | I86, 93 | ZMUC 132130 |  | GU129670 | [11] |
| Charadriiformes | Laridae | *Xema sabini* | Sabine’s gull | FI**I**CVF**C**IS**I**V | UVS | I86, 93 | LSUMZ B-4066 |  | GU129671 | [11] |
| Charadriiformes | Laridae | *Chroicocephalus ridibundus* | Black-headed gull | FI**I**CVL**C**IS**I**V | UVS | I86, 93 |  |  | AY960711 | [7] |
| Charadriiformes | Laridae | *Chroicocephalus hartlaubii* | Hartlaub’s gull | FI**I**CVL**C**IS**I**V | UVS | I86, 93 |  |  | AY960709 | [7] |
| Charadriiformes | Laridae | *Rhodostethia rosea* | Ross’s gull | FV**I**CVF**C**IS**L**V | UVS | I86, L93 | ZMUC 139640 |  | GU129669 | [11] |
| Charadriiformes | Laridae | *Leucophaeus pipixcan* | Franklin’s gull | FI**I**CVF**C**IS**I**V | UVS | I86, 93 | UWBM 80595 | CSW 6795 | GU129668 | [11] |
| Charadriiformes | Laridae | *Ichthyaetus hemprichii* | Sooty gull | FI**I**CVF**C**IS**I**V | UVS | I86, 93 |  |  | AY960710 | [7] |
| Charadriiformes | Laridae | *Larus atlanticus* | Olrog’s gull | FI**I**CVF**C**IS**I**V | UVS | I86, 93 | P Yorio, J-M Pons |  | GU129667 | [11] |
| Charadriiformes | Laridae | *Larus marinus* | Great black-backed gull | FI**I**CVF**C**IS**I**V | UVS | I86, 93 |  |  | AY227162 | [2] |
| Charadriiformes | Laridae | *Larus dominicanus* | Kelp gull | FI**I**CVF**C**IS**I**V | UVS | I86, 93 |  |  |  | [5] |
| Charadriiformes | Laridae | *Larus argentatus* 16 | European herring gull | FI**I**CVF**C**IS**I**V | UVS | I86, 93 |  |  | AY22716, FJ790324 | [2,16] |
| Charadriiformes | Laridae | *Larus michahellis* 16 | Yellow-legged gull | FI**I**CVF**C**IS**I**V | UVS | I86, 93 |  |  | FJ790325 | [12] |
| Charadriiformes | Laridae | *Larus fuscus* | Lesser black-backed Gull | FI**I**CVF**C**IS**I**V | UVS | I86, 93 |  |  | AY227161 | [2] |
| Charadriiformes | Laridae | *Hydroprogne caspia* | Caspian tern | FV**T**CIF**S**IF**T**V | VS | T86 |  |  |  | [5] |
| Charadriiformes | Laridae | *Thalasseus sandvicensis* | Sandwich tern | FV**T**CIF**S**IF**T**V | VS | T86 |  |  | AY960720 | [7] |
| Charadriiformes | Laridae | *Sternula albifrons* | Little tern | FV**T**CIF**S**IF**T**V | VS | T86 | NRM 20076398 |  | GU129679 | [11] |
| Charadriiformes | Laridae | *Onychoprion anaethetus* | Bridled tern | FV**T**CVF**S**IF**T**V | VS | T86 | ZMUC 112708 |  | GU129676 | [11] |
| Charadriiformes | Laridae | *Onychoprion fuscatus* | Sooty tern | FV**T**CVF**S**IF**T**V | VS | T86 | ZMUC 113320 |  | GU129677 | [11] |
| Charadriiformes | Laridae | *Sterna striata* | White-fronted tern | FV**T**CIF**S**IF**T**V | VS | T86 |  |  |  | [5] |
| Charadriiformes | Laridae | *Sterna hirundo* | Common tern | FV**T**CIF**S**IF**T**V | VS | T86 | Dept Animal Ecology, UU | 031218-9 | GU129682 | [11] |
| Charadriiformes | Laridae | *Sterna paradisaea* | Arctic tern | FV**T**CIF**S**IF**T**V | VS | T86 |  |  | AY960719 | [7] |
| Charadriiformes | Laridae | *Chlidonias hybrida* | Whiskered tern | FV**T**CIF**S**IF**T**V | VS | T86 | ZMUC 131916 |  | GU129681 | [11] |
| Charadriiformes | Laridae | *Chlidonias niger* | Black tern | FV**T**CIF**S**IF**T**V | VS | T86 | NRM 20066936 |  | GU129680 | [11] |
| Charadriiformes | Stercorariidae | *Stercorarius maccormicki* | South Polar skua | FV**A**CIF**S**VF**T**V | VS |  | ZMUC 131807 |  | GU129685 | [11] |
| Charadriiformes | Stercorariidae | *Stercorarius antarcticus lonnbergi* | Brown skua | FV**A**CIF**S**VF**T**V | VS |  |  |  |  | [5] |
| Charadriiformes | Stercorariidae | *Stercorarius parasiticus* 2 | Parasitic jaeger | FV**A**CIF**S**VF**T**V | VS |  | NRM 20066101, – |  | GU129684, – | [13,5] |
| Charadriiformes | Stercorariidae | *Stercorarius longicaudus* | Long-tailed jaeger | FV**A**CIF**S**VF**T**V | VS |  | NRM 976537 |  | GU129683 | [11] |
| Charadriiformes | Alcidae | *Uria aalge* | Common Murre | FL**A**CIF**S**VF**T**V | VS |  | IBG, UU |  | AY227163 | [2] |
| Charadriiformes | Alcidae | *Alca torda* | Razorbill | FV**A**CIF**S**VF**T**V | VS |  |  |  | AY227159 | [2] |
| Pteroclidiformes | Pteroclididae | *Syrrhaptes paradoxus* 2 | Pallas's sandgrouse | FI**F**CTF**S**VF**T**V | UVS |  | UWBM 59840, UWBM 59842 | CSW 5807, CSW 5809 | HF565344, HF565345 | This study |
| Pteroclidiformes | Pteroclididae | *Pterocles bicinctus* | Double-banded sandgrouse | FI**F**CSF**S**VF**T**V | UVS |  | UWBM 53231 | SVD 896 | HF565346 | This study |
| Columbiformes | Columbidae | *Columba livia* | Common pigeon | FI**S**CIF**S**VF**T**V | VS |  |  |  | AF149237, AY227168 | [14,02] |
| Columbiformes | Columbidae | *Ptilinopus magnificus* | Wompoo fruit dove | FI**S**CIF**S**VF**T**V | VS |  | O.73263.001 |  | HF565347 | This study |
| Psittaciformes | Strigopidae | *Nestor notabilis* 3 | Kea | FL**A**CIF**C**IF**T**V | UVS |  |  | –, Nn, NnB | HM150807HM222548, HM222549 | [15,1] |
| Psittaciformes | Strigopidae | *Nestor meridionalis* 2 | New Zealand kaka | FL**A**CIF**C**IF**T**V | UVS |  |  | Nm, NmB | HM222548, HM222549 | [1] |
| Psittaciformes | Strigopidae | *Strigops habroptilus* | Kakapo | FL**A**CIF**C**IF**T**V | UVS |  |  | Sh | HM222552 | [1] |
| Psittaciformes | Cacatuidae | *Calyptorhynchus latirostris* | Short-billed black cockatoo | FL**A**CIF**C**IF**T**V | UVS |  |  |  | HM150800 | [15] |
| Psittaciformes | Cacatuidae | *Eolophus roseicapilla* 2 | Galah | FL**A**CIF**C**IF**T**V | UVS |  |  | –, 6-Er | HM150801, HM222554 | [15,1] |
| Psittaciformes | Cacatuidae | *Cacatua galerita* 2 | Sulphur-crested cockatoo | FL**A**CIF**C**IF**T**V | UVS |  |  | –, CgB | HM150802, HM222556, | [15,1] |
| Psittaciformes | Cacatuidae | *Cacatua alba* | White cockatoo | FL**A**CIF**C**IF**T**V | UVS |  | Djurkliniken Roslagstull, Stockholm |  | HF565348 | This study |
| Psittaciformes | Cacatuidae | *Cacatua moluccensis* 2 | Salmon-crested Cockatoo | FL**A**CIF**C**IF**T**V | UVS |  |  |  | HM150797, HM150798 | [15] |
| Psittaciformes | Cacatuidae | *Nymphicus hollandicus* 2 | Cockatiel | FL**A**CIF**C**IF**T**V | UVS |  | –, Fyris Zoo, Uppsala | Nh, – | HF565349 | [1], this study |
| Psittaciformes | Cacatuidae | *Trichoglossus haematodus* | Coconut lorikeet | FL**A**CIF**C**IF**T**V | UVS |  |  | RL | HM222553 | [1] |
| Psittaciformes | Cacatuidae | *Lorius garrulus* | Chattering lory | FL**A**CIF**C**IF**T**V | UVS |  |  | 4-Lg | HM222555 | [1] |
| Psittaciformes | Psittacidae | *Barnardius zonarius semitorquatus* | Australian ringneck | FL**A**CIF**C**IF**T**V | UVS |  |  |  | HM150799 | [15] |
| Psittaciformes | Psittacidae | *Platycercus elegans* 2 | Crimson rosella | FL**A**CIF**C**IF**T**V | UVS |  |  | –, 15-Pe | HM150794, HM222560 | [15,1] |
| Psittaciformes | Psittacidae | *Platycercus eximius* | Eastern rosella | FL**X**CIF**C**IF**T**V | UVS? |  |  | Pe | HM222558 | [1] |
| Psittaciformes | Psittacidae | *Melopsittacus undulatus* 2 | Budgerigar | FL**A**CII**C**IF**T**V | UVS |  |  |  | Y11787, AY227185 | [16,2] |
| Psittaciformes | Psittacidae | *Eclectus roratus* | Eclectus parrot | FL**X**CIF**C**IF**T**X | UVS? |  |  | 7-Eror | HM222563 |  |
| Psittaciformes | Psittacidae | *Psittacula krameri manillensis* | Rose-ringed Parakeet | FL**A**CIF**C**IF**T**V | UVS |  |  | Pkm | HM222562 |  |
| Psittaciformes | Psittacidae | *Psittacula derbiana* | Lord Derby's parakeet | FL**A**CIF**C**IF**T**V | UVS |  |  | 14-Pd | HM222561 |  |
| Psittaciformes | Psittacidae | *Agapornis roseicollis* | Rosy-faced lovebird | FL**A**CIF**C**IF**T**V | UVS |  |  | 1-Ar | HM222559 |  |
| Psittaciformes | Psittacidae | *Ara ararauna* | Blue-and-yellow macaw | FL**A**CIF**C**IF**T**V | UVS |  |  |  | HM150803 | [15] |
| Psittaciformes | Psittacidae | *Ara macao* | Scarlet macao | FL**A**CIF**C**IF**T**V | UVS |  |  |  | HM150792 | [15] |
| Psittaciformes | Psittacidae | *Psittacus erithacus* | Grey parrot | FL**A**CIF**C**IF**T**V | UVS |  | IBG, UU |  | AY227186, HM150804 | [2,15] |
| Psittaciformes | Psittacidae | *Anodorhynchus hyacinthinus* | Hyacinth Macaw | FP**A**CIF**C**IF**T**V | UVS |  |  |  | HM150806 | [15] |
| Psittaciformes | Psittacidae | *Ara chloropterus* | Red-and-green macaw | FL**A**CIF**C**IF**T**V | UVS |  |  |  | HM150805 | [15] |
| Psittaciformes | Psittacidae | *Aratinga aurea* | Peach-fronted parakeet | FL**A**CIF**C**IF**T**V | UVS |  | NRM 976646 | AHN-308 | HF565350 | This study |
| Psittaciformes | Psittacidae | *Pyrrhura frontalis* | Maroon-bellied parakeet | FL**A**CIF**C**IF**T**V | UVS |  | NRM 966979 | GFK-257 | HF565351 | This study |
| Psittaciformes | Psittacidae | *Forpus xanthopterygius* | Blue-winged parrotlet | FL**A**CIF**C**IF**T**V | UVS |  | NRM 986799 | LAA-094 | HF565352 | This study |
| Psittaciformes | Psittacidae | *Amazona versicolor* | St. Lucia amazon | FL**A**CIF**C**IF**T**V | UVS |  |  |  | HM150793 | [15] |
| Psittaciformes | Psittacidae | *Amazona guildingii* | St. Vincent amazon | FL**A**CIF**C**IF**T**V | UVS |  |  |  | HM150796, HM150795 | [15] |
| Opisthocomiformes | Opisthocomidae | *Opisthocomus hoazin* | Hoatzin | FI**C**CIF**S**VF**T**V | VS |  | LSUMZ B-10753 |  | HF565353 | This study |
| Musophagiformes | Musophagidae | *Tauraco porphyreolophus* | Purple-crested turaco | FI**S**CIF**S**VF**T**V | VS |  | UWBM 52953 | SAR 6784 | HF565354 | This study |
| Cuculiformes | Cuculidae | *Urodynamis taitensis* 2 | Long-tailed cuckoo | FI**S**CIF**S**VF**T**V | VS |  | E56, E59 |  | HM159123, HM159124 | Hauber & Chong /GenBank only |
| Cuculiformes | Cuculidae | *Chrysococcyx lucidus 2* | Shining bronze cuckoo | FI**S**CIF**S**VF**T**V | VS |  | SC1, SC02 |  | HM159121, HM159122 | Hauber & Chong GenBank only |
| Caprimulgiformes | Caprimulgidae | *Caprimulgus europaeus* | European nightjar | FL**C**CVF**S**VF**T**V | VS |  | NRM 986344 |  | AY227187 | [2] |
| Apodiformes | Trochilidae | *Phaethornis pretrei* | Planalto hermit | FM**C**CIF**S**VF**T**V | VS |  | NRM 967134 |  | GQ305950 | [13] |
| Apodiformes | Trochilidae | *Hylocharis chrysura* | Gilded hummingbird | FL**C**CIF**S**VF**T**V | VS |  | NRM 996701 |  | GQ305954 | [13] |
| Apodiformes | Trochilidae | *Heliodoxa rubinoides* | Fawn-breasted brilliant | FM**C**CIF**S**IS**I**V | VS | I93 | NRM 20046023 |  | GQ305951 | [13] |
| Trogoniformes | Trogonidae | *Trogon curucui* | Blue-crowned trogon | FI**F**CVF**S**VF**T**V | UVS |  | NRM937172 | AHN-047 | AY227190 | [2] |
| Trogoniformes | Trogonidae | *Harpactes erythrocephalus* | Red-headed trogon | FI**F**CVF**S**VF**T**V | UVS |  | NRM 20026658 | VNM2002-049 | HF565355 | This study |
| Coraciiformes | Coraciidae | *Coracias garrulus* | European roller | FI**S**CIF**S**VF**T**V | VS |  | NRM 857339 |  | AY227170 | [2] |
| Coraciiformes | Alcedinidae | *Alcedo atthis* | Common kingfisher | FI**S**CIF**S**VF**T**V | VS |  | Dept Animal Ecology, UU |  | AY227169 | [2] |
| Coraciiformes | Momotidae | *Momotus momota* | Amazonian motmot | FI**F**CSF**S**VF**T**V | UVS |  | NRM 947281 | ICM-078 | HF565356 | This study |
| Coraciiformes | Momotidae | *Baryphthengus ruficapillus* | Rufous-capped motmot | FI**F**CSF**S**VF**T**V | UVS |  | NRM 937319 |  | HF565357 | This study |
| Coraciiformes | Momotidae | *Eumomota superciliosa* | Turquoise-browed motmot | FI**F**CSF**S**VF**T**V | UVS |  | NRM 20066359 |  | HF565358 | This study |
| Coraciiformes | Meropidae | *Merops apiaster* | European bee-eater | FV**S**CIF**S**VF**T**V | VS |  | S Berlin, Dept Evolutionary Biology, UU | "1/1" | HF565359 | This study |
| Bucerotiformes | Upupidae | *Upupa epops* | Eurasian hoopoe | FM**S**CIF**S**VF**T**V | VS |  | NRM 966502 |  | AY227191 | [2] |
| Piciformes | Bucconidae | *Nystalus maculatus* | Caatinga puffbird | FI**S**CIF**S**VF**T**V | VS |  | NRM 947240 | ICM-055 | AY227171 | [2] |
| Piciformes | Megalaimidae | *Megalaima virens* | Great barbet | FI**S**CIF**S**VF**T**V | VS |  | R den Tex, Dept Evolutionary Biology, UU | X-2009 | HF565360 | This study |
| Piciformes | Ramphastidae | *Ramphastos tucanus* | White-throated toucan | FI**S**CIF**S**VF**T**V | VS |  |  | Tukan | HF565361 | This study |
| Piciformes | Picidae | *Dendrocopos major* | Great spotted woodpecker | FL**S**CIF**S**VF**T**V | VS |  | IBG, UU |  | AY227184 | [2] |
| Piciformes | Picidae | *Picus viridis* | European green woodpecker | FL**S**CIF**S**VF**T**V | VS |  | AÖ | Sko01 | HF565362 | This study |
| Passeriformes | Acanthisittidae | *Acanthisitta chloris* | Rifleman | LL**C**CIL**C**VF**L**V | UVS | L93 | AJ Baker | 6 | HE601812 | [13] |
| Passeriformes | Pittidae | *Hydrornis elliotii* | Bar-bellied pitta | FL**C**CIF**S**VF**T**V | VS |  | NRM 200470000 |  | HE601813 | [13] |
| Passeriformes | Thamnophilidae | *Hypocnemis peruviana* | Peruvian warbling antbird | FL**C**CIF**S**VF**T**V | VS |  | CICRA, N Seddon | 29.09.07 | GQ924590 | [17] |
| Passeriformes | Thamnophilidae | *Myrmeciza hemimelaena* | Southern chestnut-tailed antbird | FL**C**CIF**S**VF**T**V | VS |  | CICRA, N Seddon | 22.11.07 | GQ924591 | [17] |
| Passeriformes | Thamnophilidae | *Phlegopsis nigromaculata* | Black-spotted bare-eye | FL**C**CIF**S**VF**T**V | VS |  | CICRA, N Seddon | 10.11.04 | GQ924592 | [17] |
| Passeriformes | Tyrannidae | *Camptostoma obsoletum* | Southern beardless tyrannulet | FM**C**CIF**S**VF**T**V | VS |  | NRM 937368 | CMS-018 | HE601814 | [13] |
| Passeriformes | Tyrannidae | *Sayornis phoebe* | Eastern phoebe | FM**C**CIF**S**VF**T**X | VS |  |  |  |  | [1] |
| Passeriformes | Tyrannidae | *Xolmis irupero* | White monjita | FM**C**CIF**S**VF**T**V | VS |  | NRM 937384 | ICM-033 | HE601815 | [13] |
| Passeriformes | Tyrannidae | *Tyrannus savana* | Fork-tailed flycatcher | FM**C**CIF**S**VF**T**V | VS |  | NRM 937164 | AHN-039 | HE601816 | [13] |
| Passeriformes | Tyrannidae | *Tyrannus tyrannus* | Eastern kingbird | FM**C**CIF**S**VF**T**X | VS |  |  |  |  | [1] |
| Passeriformes | Tyrannidae | *Myiarchus tyrannulus* | Brown-crested flycatcher | FM**C**CIF**S**VF**T**V | VS |  | NRM 937173 | AHN-048 | AY227183 | [2] |
| Passeriformes | Pipridae | *Manacus manacus* | White-bearded manakin | FM**C**CIF**S**VF**T**X | VS |  |  |  |  | [1] |
| Passeriformes | Pipridae | *Manacus manacus* 2 | White-bearded manakin | FM**C**CIF**S**VF**T**V | VS |  | L Shorey, Dept Population Biology, UU | LS015, LS241 | HF565363, AY227182 | This study, this study reanalysed from [2] |
| Passeriformes | Tityridae | *Onychorhynchus coronatus* | Amazonian royal flycatcher | FF**C**CIF**S**VF**V**V | VS |  | UWBM 56019 | DAB 1082 | HE601817 | [13] |
| Passeriformes | Menuridae | *Menura alberti* | Albert's lyrebird | FF**C**CIF**C**VF**T**V | UVS |  | ANWC47113 |  | HE601818 | [13] |
| Passeriformes | Menuridae | *Menura novaehollandiae* | Superb lyrebird | FF**C**CIF**C**VF**T**V | UVS |  | O.70813 | EBU 37068 | HE601819 | [13] |
| Passeriformes | Ptilonorhynchidae | *Ailuroedus crassirostris* | Green catbird | FL**C**CIF**S**VF**T**V | VS |  | O.71114 | EBU 39507 | HE588090 | [18] |
| Passeriformes | Ptilonorhynchidae | *Sericulus chrysocephalus* | Regent bowerbird | FL**C**CIF**S**VF**T**V | VS |  | O.70804 | EBU 38490 | HE588091 | [18] |
| Passeriformes | Ptilonorhynchidae | *Chlamydera nuchalis* | Great bowerbird | FL**C**CIF**S**VF**T**V | VS |  | O.65796 | EBU 10352 | HE588092 | [18] |
| Passeriformes | Maluridae | *Chenorhamphus grayi* | Broad-billed fairywren | FL**C**CIF**S**VF**T**V | VS |  | KUNHM 97993 | 7082 | HE588093 | [18] |
| Passeriformes | Maluridae | *Malurus cyanocephalus* | Emperor fairywren | FL**C**CIF**S**VF**T**V | VS |  | KUNHM 87988 | 7564 | HE588094 | [18] |
| Passeriformes | Maluridae | *Malurus amabilis* | Lovely fairywren | FL**C**CIF**C**IF**T**V | UVS |  | S Pruett-Jones | LW01A | HE588095 | [18] |
| Passeriformes | Maluridae | *Malurus lamberti lamberti* | Variegated fairywren | FL**C**CIF**C**IF**T**V | UVS |  | ANWC 46335 | 46335 | HE588097 | [18] |
| Passeriformes | Maluridae | *Malurus lamberti assimilis* | Purple-backed fairywren | FL**C**CIF**C**IF**T**V | UVS |  | S Pruett-Jones | VW104 | HE588096 | [18] |
| Passeriformes | Maluridae | *Malurus pulcherrimus* | Blue-breasted fairywren | XL**C**CIF**C**IF**T**V | UVS |  | UWBM 60862 | PLG300 | HE588098 | [18] |
| Passeriformes | Maluridae | *Malurus elegans* | Red-winged fairywren | FL**C**CIF**C**IF**T**V | UVS |  | WAM A27629 | A27629 | HE588099 | [18] |
| Passeriformes | Maluridae | *Malurus cyaneus* | Superb fairywren | FL**C**CIF**C**IF**T**V | UVS |  | ANWC B34618 |  | HE588100 | [18] |
| Passeriformes | Maluridae | *Malurus splendens musgravi* | Turquoise fairywren | FL**C**CIF**C**IF**T**V | UVS |  | ANSP 189635 | 10547 | HE588101 | [18] |
| Passeriformes | Maluridae | *Malurus splendens emmottorum* | Ssp of splendid fairywren | FL**C**CIF**C**IF**T**V | UVS |  | UWBM 57545 | SAR7063 | HE588102 | [18] |
| Passeriformes | Maluridae | *Malurus splendens melanotus* 5 | Black-backed fairywren | FL**C**CIF**C**IF**T**V | UVS |  | S Pruett-Jones | SW256, -268, -683 -727, -736 | HE588103-HE588107 | [18] |
| Passeriformes | Maluridae | *Malurus coronatus* 2 | Purple-crowned fairywren | FL**C**CIF**S**VF**T**V | VS |  | S Pruett-Jones | 2003–01, 2003–02 | HE588108, HE588109 | [18] |
| Passeriformes | Maluridae | *Malurus alboscapulatus naimii* | White-shouldered fairywren | FL**C**CIF**S**VF**T**V | VS |  | ANWC B26769 | MVM E121 | HE588110 | [18] |
| Passeriformes | Maluridae | *Malurus melanocephalus* 3 | Red-backed fairywren | FL**C**CIF**S**VF**T**V | VS |  | ANWC B29594, B50894, J Karubian | –, –, RBW01 | HE588111-HE588113 | [18] |
| Passeriformes | Maluridae | *Malurus leucopterus edouardi* 3 | Ssp of white-winged fairywren | FL**C**CIF**S**VF**T**V | VS |  | S Pruett-Jones | BW008, BW013, BW015 | HE588114-HE588116 | [18] |
| Passeriformes | Maluridae | *Malurus leucopterus leuconotus* 2 | Blue-and-white fairywren | FL**C**CIF**S**VF**T**V | VS |  | S Pruett-Jones | WW10, WW14 | HE588117, HE588118 | [18] |
| Passeriformes | Maluridae | *Malurus leucopterus leucopterus* 2 | Black-and-white fairywren | FL**C**CIF**S**VF**T**V | VS |  | WAM A26803, -26804, -26806 | A26803, A26804, A26806 | HE588119, HE588120 | [18] |
| Passeriformes | Maluridae | *Clytomyias insignis* | Orange-crowned fairywren | FL**C**CIF**S**VF**T**V | VS |  |  | Kan4619 | HE588121 | [18] |
| Passeriformes | Maluridae | *Stipiturus mallee* | Mallee emuwren | FL**C**CIF**S**VF**T**V | VS |  | S Pruett-Jones | MEW1 | HE588122 | [18] |
| Passeriformes | Maluridae | *Amytornis barbatus* | Grey grasswren | FL**C**CIF**S**VF**T**V | VS |  | ANWC 41788 |  | HE588123 | [18] |
| Passeriformes | Maluridae | *Amytornis striatus* | Striated grasswren | FL**C**CIF**S**VS**T**V | VS |  | S Pruett-Jones | SGW1 | HE588124 | [18] |
| Passeriformes | Meliphagidae | *Lichenostomus flavescens* | Yellow-tinted honeyeater | FM**C**CIF**S**VF**T**V | VS |  | O.66338 | EBU 10401 | GQ305955 | [13] |
| Passeriformes | Meliphagidae | *Philemon argenticeps* | Silver-crowned friarbird | FM**C**CIF**S**VF**T**V | VS |  | O.65825 | EBU 10283 | GQ305956 | [13] |
| Passeriformes | Meliphagidae | *Phylidonyris novaehollandiae* | New Holland honeyeater | FM**C**CIF**S**VF**T**V | VS |  | O.70854 | EBU 38188 | GQ305957 | [13] |
| Passeriformes | Meliphagidae | *Conopophila rufogularis* | Rufous-throated honeyeater | FM**C**CIF**S**VF**T**V | VS |  | O.65927 | EBU 10376 | GQ305958 | [13] |
| Passeriformes | Meliphagidae | *Acanthorhynchus tenuirostris* | Eastern spinebill | FM**C**CIF**S**VF**T**V | VS |  | O.70913 | EBU 38146 | GQ305959 | [13] |
| Passeriformes | Acanthizidae | *Gerygone igata* 2 | Grey gerygone | FM**C**CIF**S**VF**T**V | VS |  | GW1, GW2 |  | HM159130, HM159131 | [1] |
| Passeriformes | Family Incertae Sedis | *Mohoua albicilla* | Whitehead | LM**C**CIF**C**LF**T**X | UVS |  |  |  |  | [1] |
| Passeriformes | Pomatostomidae | *Pomatostomus temporalis* | Grey-crowned babbler | FL**C**CIF**S**VF**T**V | VS |  | LSUMZ-B23626 |  | HE601820 | [10] |
| Passeriformes | Pomatostomidae | *Pomatostomus ruficeps* | Chestnut-crowned babbler | FL**C**CIF**S**VF**T**V | VS |  | LSUMZ-B23409 |  | HE601821 | [10] |
| Passeriformes | Orthonychidae | *Orthonyx temminckii* | Australian logrunner | FF**C**CIF**C**VF**T**V | UVS |  | UWBM 76694 | WBJ 3019 | HE601822 | [10] |
| Passeriformes | Cnemophilidae | *Cnemophilus loriae* | Loria's satinbird | LM**C**CIF**S**VF**T**V | VS |  | NRM 569572 |  | HE601823 | [10] |
| Passeriformes | Melanocharitidae | *Toxorhamphus poliopterus* | Slaty-headed longbill | LM**C**CVF**C**IF**T**V | UVS |  | NRM 543574 |  | HE601824 | [10] |
| Passeriformes | Callaeidae | *Philesturnus carunculatus* | South Island saddleback | LM**C**CVF**C**IF**T**V | UVS |  | PHCA |  | HM159129 | Hauber & Chong GenBank only |
| Passeriformes | Campephagidae | *Coracina novaehollandiae* | Black-faced cuckooshrike | FL**C**CIF**S**VF**T**V | VS |  | ANWC 50378 |  | HE601825 | [10] |
| Passeriformes | Vireonidae | *Cyclarhis gujanensis* | Rufous-browed peppershrike | LM**C**CVF**C**IF**T**V | UVS |  | UWBM 77444 | VGR 283 | HE601826 | [10] |
| Passeriformes | Vireonidae | *Vireo belli* | Bell's vireo | LM**C**CVF**C**IF**T**V | UVS |  | UWBM 81347 | VGR 877 | HE601827 | [10] |
| Passeriformes | Oriolidae | *Oriolus oriolus* | Eurasian golden oriole | FL**C**CIF**S**VF**T**V | VS |  | NRM 20036563 |  | HE601828 | [10] |
| Passeriformes | Dicruridae | *Dicrurus bracteatus* | Spangled drongo | FL**C**CIF**S**VF**T**V | VS |  | ANWC 29889 |  | HE601829 | [10] |
| Passeriformes | Rhipiduridae | *Rhipidura leucophrys* | Willie wagtail | FL**C**CIF**S**VF**T**V | VS |  | ANWC 32827 |  | HE601830 | [10] |
| Passeriformes | Rhipiduridae | *Rhipidura albiscapa* | Grey fantail | FL**C**CIF**S**VF**T**V | VS |  | ANWC 50231 |  | HE601831 | [10] |
| Passeriformes | Corvidae | *Cyanocorax chrysops* | Plush-crested jay | FL**C**CIF**S**VF**T**V | VS |  | NRM 956690 | GFK-178 | HE601832 | [10] |
| Passeriformes | Corvidae | *Garrulus glandarius* | Eurasian jay | FL**C**CIF**S**VF**T**V | VS |  | P Halvarsson, Dept Population Biology, UU | 6188056 | HE601833 | [10] |
| Passeriformes | Corvidae | *Pica pica* | Eurasian magpie | FL**C**CIF**S**VF**T**V | VS |  | Uppsala kommun/Dept Animal Ecology UU | Hov17 | GQ305970 | [46] |
| Passeriformes | Corvidae | *Coloeus monedula* | Western jackdaw | FL**C**CIF**S**VF**T**V | VS |  |  |  | AY227177 | [2] |
| Passeriformes | Corvidae | *Corvus frugilegus* | Rook | FL**C**CIF**S**VF**T**V | VS |  |  |  | DQ451006 | Browne et al. Genbank only |
| Passeriformes | Corvidae | *Corvus cornix* | Hooded crow | FM**C**CIF**S**VF**T**V | VS |  |  |  | AY227176 | [2] |
| Passeriformes | Paradisaeidae | *Manucodia comrii* | Curl-crested manucode | FL**C**CIF**S**VF**T**V | VS |  | O.60598 | EBU 10071 | HE601834 | [10] |
| Passeriformes | Paradisaeidae | *Ptiloris magnificus* | Magnificent riflebird | FL**C**CIF**S**VF**T**V | VS |  | O.64926 | EBU 11365 | HE601835 | [10] |
| Passeriformes | Paradisaeidae | *Paradisaea rudolphi* | Blue bird-of-paradise | FM**C**CIF**S**VF**X**V | VS |  | NRM 20046265 | Gelin-N28 | HE601836 | [10] |
| Passeriformes | Petroicidae | *Eopsaltria australis* | Eastern yellow robin | LM**C**CIF**C**LF**T**V | UVS |  | O.71121 | EBU 39591 | HE601837 | [10] |
| Passeriformes | Petroicidae | *Microeca fascinans* | Jacky winter | LM**C**CIF**C**LF**T**V | UVS |  | O.65957 | EBU 10407 | HE601838 | [10] |
| Passeriformes | Petroicidae | *Petroica rosea* | Rose robin | LM**C**CIF**C**LF**T**V | UVS |  | O.70044 | EBU 10234 | HE601839 | [10] |
| Passeriformes | Petroicidae | *Petroica goodenovii* 2 | Red-capped robin | LM**C**CIF**C**LF**T**V | UVS |  | D Dowling | 274, 278 | HE601840, HE601841 | [10] |
| Passeriformes | Paridae | *Cyanistes caeruleus* | Eurasian blue tit | LM**C**CVF**C**IF**T**V | UVS |  | N Backström & S Berlin | BT49 | FJ440638 | [10] |
| Passeriformes | Pycnonotidae | *Pycnonotus cafer* | Red-vented bulbul | LM**M**CIF**C**IF**T**V | UVS |  | NRM 20036334 |  | HE601842 | [10] |
| Passeriformes | Hirundinidae | *Hirundo rustica* | Barn swallow | LM**M**CIF**C**IF**T**V | UVS |  | T Sirotkin, AÖ | Fi15 | HE601843 | [10] |
| Passeriformes | Phylloscopidae | *Phylloscopus trochilus* | Willow warbler | LM**M**CIF**C**IF**T**V | UVS |  | M Vila-Taboada, AÖ | Ups01 | AY227181 | [2] |
| Passeriformes | Acrocephalidae | *Acrocephalus stentoreus* | Clamorous reed warbler | LM**M**CI**F**CIF**T**V | UVS |  | C Hemborg & AÖ | Eg10 | HE601844 | [10] |
| Passeriformes | Acrocephalidae | *Acrocephalus schoenobaenus* | Sedge Warbler | LM**M**CI**F**CIF**T**V | UVS |  | M Wilson, C Hemborg, S Ulfstrand, AÖ | Ug51 | HE601845 | [10] |
| Passeriformes | Acrocephalidae | *Acrocephalus scirpaceus fuscus* | Eurasian reed warbler | LM**M**CI**F**CIF**T**V | UVS |  | M Wilson, C Hemborg, S Ulfstrand, AÖ | Ug49 | HE601846 | [10] |
| Passeriformes | Acrocephalidae | *Hippolais polyglotta* | Melodious warbler | LM**M**CI**F**CIF**T**V | UVS |  | A Lindström, AÖ | It20 | HE601847 | [10] |
| Passeriformes | Donacobiidae | *Donacobius atricapilla* | Black-capped donacobius | LM**M**CIF**C**IF**T**V | UVS |  | NRM 966966 | GFK-244 | HE601848 | [10] |
| Passeriformes | Timaliidae | *Leiothrix lutea* | Red-billed leiothrix | LM**M**CVF**C**IF**T**V | UVS |  | NRM 20026687 | VNM 2002-078 | FJ440645 | [3] |
| Passeriformes | Zosteropidae | *Zosterops japonicus* | Japanese white-eye | LM**M**CIF**C**IF**T**V | UVS |  | NRM 20026678 |  | GQ305960 | [13] |
| Passeriformes | Zosteropidae | *Zosterops senegalensis* | African yellow white-eye | LM**M**CIF**C**IF**T**V | UVS |  | NRM 20066253 |  | GQ305961 | [13] |
| Passeriformes | Promeropidae | *Promerops gurneyi* | Gurney’s sugarbird | LM**C**CVF**C**IF**T**V | UVS |  | UWBM 70395 | GAV 447 | GQ305965 | [13] |
| Passeriformes | Regulidae | *Regulus regulus* | Goldcrest | LM**C**CIF**C**IF**T**V | UVS |  | Dept Animal Ecology, UU | 7.030218 | HE601849 | [10] |
| Passeriformes | Troglodytidae | *Troglodytes aedon* | House wren | LM**C**CIF**C**IF**T**V | UVS |  | N Backström, S Berlin | E3 | HE601850 | [10] |
| Passeriformes | Sittidae | *Sitta europaea 2* | Eurasian nuthatch | LM**C**CIF**C**IF**T**V | UVS |  | J Tomiuk | Kl207, Kl241 | HE601851, HE601852 | [10] |
| Passeriformes | Mimidae | *Dumetella carolinensis* | Grey catbird | LM**C**CVF**C**IF**T**X | UVS |  |  |  |  | [1] |
| Passeriformes | Mimidae | *Mimus polyglottos* | Northern mockingbird | LM**C**CVF**C**IF**T**X | UVS |  |  |  |  | [1] |
| Passeriformes | Mimidae | *Mimus saturninus* | Chalk-browed mockingbird | LM**C**CIF**C**IF**T**V | UVS |  | NRM 966912 | PER-190 | GQ305972 | [19] |
| Passeriformes | Sturnidae | *Acridotheres tristis* | Common myna | LM**C**CIF**C**IF**T**V | UVS |  | NRM 20046711 |  | HE601853 | [10] |
| Passeriformes | Sturnidae | *Sturnus vulgaris* | Common starling | LM**C**CIF**C**IF**T**V | UVS |  | A Lindström, AÖ | It16 | AY227180 | [2] |
| Passeriformes | Turdidae | *Hylocichla mustelina* | Wood thrush | LM**C**CVF**C**IF**T**X | UVS |  |  |  |  | [1] |
| Passeriformes | Turdidae | *Turdus merula* | Common blackbird | LM**C**CVF**C**IF**T**V | UVS |  | N Backström, S Berlin | TuMe2 | FJ440637 | [3] |
| Passeriformes | Turdidae | *Turdus iliacus* | Redwing | LM**C**CVF**C**IF**T**V | UVS |  | M Vila-Taboada, AÖ | Ups02 | HE601854 | [10] |
| Passeriformes | Turdidae | *Turdus migratorius* | American robin | FM**C**CVF**C**IF**T**X | UVS |  |  |  |  | [1] |
| Passeriformes | Muscicapidae | *Luscinia svecica* | Bluethroat | LM**C**CVF**C**IF**T**V | UVS |  | A Lindström, AÖ | Fi21 | HE601855 | [10] |
| Passeriformes | Muscicapidae | *Muscicapa aquatica* | Swamp flycatcher | LM**C**CVF**C**IF**T**V | UVS |  | M Wilson, C Hemborg, S Ulfstrand, AÖ | Ug40 | HE601856 | [10] |
| Passeriformes | Muscicapidae | *Ficedula hypoleuca* | European pied flycatcher | LM**C**CVF**C**IF**T**V | UVS |  | A Qvarnström | Kol36 | HE601857 | [10] |
| Passeriformes | Muscicapidae | *Ficedula albicollis* | Collared flycatcher | LM**C**CVF**C**IF**T**V | UVS |  | A Qvarnström | Kol6 | HE601858 | [10] |
| Passeriformes | Passeridae | *Passer domesticus* | House sparrow | LM**C**CVF**C**IF**T**X |  |  |  |  |  | [1] |
| Passeriformes | Nectariniidae | *Chalcomitra senegalensis* | Scarlet-chested sunbird | LM**C**CVF**C**IF**T**V | UVS |  | NRM 20056357 |  | GQ305963 | [13] |
| Passeriformes | Nectariniidae | *Cinnyris pulchellus* | Beautiful sunbird | LM**C**CVF**C**IF**T**V | UVS |  | NRM 20076163 |  | GQ305964 | [13] |
| Passeriformes | Nectariniidae | *Aethopyga siparaja* | Crimson sunbird | LM**C**CVF**C**IF**T**V | UVS |  | NRM 20026613 |  | GQ305962 | [13] |
| Passeriformes | Estrildidae | *Amadina fasciata* | Cut-throat finch | LM**C**CVF**C**IF**T**V | UVS |  | ZMUC 118505 |  | FJ440639 | [3] |
| Passeriformes | Estrildidae | *Neochmia modesta* | Plum-headed finch | LM**C**CVF**C**IF**T**V | UVS |  | ANWC 29034 |  | FJ440642 | [3] |
| Passeriformes | Estrildidae | *Taeniopygia guttata* | Zebra finch | LM**C**CVF**C**IF**T**V | UVS |  |  |  | AF222331 | [6] |
| Passeriformes | Estrildidae | *Erythrura gouldiae* | Gouldian finch | LM**C**CVF**C**IF**T**V | UVS |  |  | EBU 11103 | FJ440640 | [3] |
| Passeriformes | Estrildidae | *Lonchura maja* | White-headed munia | LM**C**CVF**C**IF**T**V | UVS |  | ZMUC 118479 |  | FJ440641 | [3] |
| Passeriformes | Motacillidae | *Motacilla flava* 17 | Western yellow wagtail | LM**C**CVF**C**IF**T**V | UVS |  | Various sources |  | HE601859 | [10] |
| Passeriformes | Motacillidae | *Motacilla citreola* | Citrine wagtail | LM**C**CVF**C**IF**T**V | UVS |  | P Chylarecki | M.cit.3 | HE601860 | [10] |
| Passeriformes | Motacillidae | *Motacilla cinerea* | Grey wagtail | LM**C**CVF**C**IF**T**V | UVS |  | T Sirotkin, AÖ | Var03 | HE601861 | [10] |
| Passeriformes | Motacillidae | *Motacilla alba* | White wagtail | LM**C**CVF**C**IF**T**V | UVS |  | C Hemborg | 3 | HE601862 | [10] |
| Passeriformes | Motacillidae | *Motacilla aguimp* | African pied wagtail | LM**C**CVF**C**IF**T**V | UVS |  | M Wilson, AÖ | Ug01 | HE601863 | [10] |
| Passeriformes | Motacillidae | *Macronyx croceus* | Yellow-throated longclaw | XM**C**CVF**C**IF**T**V | UVS |  | M Wilson, C Hemborg, S Ulfstrand, AÖ | Ug59 | HE601864 | [10] |
| Passeriformes | Motacillidae | *Anthus cervinus* | Red-throated pipit | LM**C**CVF**C**IF**T**V | UVS |  | M Björklund, AÖ | Is09 | HE601865 | [10] |
| Passeriformes | Fringillidae | *Serinus canaria* | Atlantic canary | LM**C**CVF**C**IF**T**V | UVS |  |  |  | –, AJ277922 | [20,21] |
| Passeriformes | Fringillidae | *Himatione sanguinea* | ’Apapane | LM**C**CVF**C**IF**T**V | UVS |  | UWBM 65834 | WLK 133 | GQ305968 | [13] |
| Passeriformes | Parulidae | *Dendroica petechia* | Mangrove warbler | LM**C**CVF**C**IF**T**X | UVS |  |  |  |  | [1] |
| Passeriformes | Parulidae | *Dendroica coronata* | Myrtle warbler | LM**C**CVF**C**IF**T**V | UVS |  | FMNH 442959 |  | HE601866 | [10] |
| Passeriformes | Icteridae | *Icterus galbula* | Baltimore oriole | LM**C**CVF**C**IF**T**V | UVS |  | FMNH 442567 |  | GQ305968 | [13] |
| Passeriformes | Icteridae | *Molothrus ater* | Brown-headed cowbird | LM**C**CVF**C**IF**T**X | UVS |  |  |  |  | [1] |
| Passeriformes | Icteridae | *Agelaius phoeniceus* | Red-winged blackbird | LM**C**CVF**C**IF**T**X | UVS |  |  |  |  | [1] |
| Passeriformes | Icteridae | *Quiscalus quiscula* | Common grackle | LM**C**CVF**C**IF**T**X | UVS |  |  |  |  | [1] |
| Passeriformes | Icteridae | *Sturnella superciliaris* | White-browed blackbird | LM**C**CVF**C**IF**T**V | UVS |  | FMNH 330789 |  | HE601867 | [10] |
| Passeriformes | Icteridae | *Sturnella neglecta* | Western meadowlark | LM**C**CVF**C**IF**T**V | UVS |  | FMNH 341967 |  | HE601868 | [10] |
| Passeriformes | Icteridae | *Xanthocephalus xanthocephalus* | Yellow-headed blackbird | LM**C**CVF**C**IF**T**V | UVS |  | FMNH 442337 |  | HE601869 | [10] |
| Passeriformes | Icteridae | *Dolichonyx oryzivorus* 2 | Bobolink | LM**C**CVF**C**IF**T**V | UVS |  | UWBM 80613, -80615 | EVL 750, EVL752 | FJ440643 | [3] |
| Passeriformes | Emberizidae | *Emberiza citrinella* | Yellowhammer | LM**C**CVF**C**IF**T**V | UVS |  | NRM 986194 |  | GQ305971 | [19] |
| Passeriformes | Emberizidae | *Melospiza melodia* | Song sparrow | LM**C**CVF**C**IF**T**X | UVS |  |  |  |  | [1] |
| Passeriformes | Thraupidae | *Cyanerpes cyaneus* | Red-legged honeycreeper | LM**C**CVF**C**IF**T**V | UVS |  | FMNH 391637 |  | GQ305966 | [13] |

Notes: *Academy of Natural Sciences, Philadelphia (ANSP), Australian Museum (O., EBU), Australian National Wildlife Collection (ANWC), Biology Education Centre, Uppsala university (IBG), Centro de Investigación y Conservación de Río Los Amigos, Madre de Dios, Peru (CICRA), Field Museum of Natural History, Chicago (FMNH), Louisiana State University Museum of Natural Science, Baton Rouge (LSUMZ), National Veterinary Institute of Sweden (SVA), Swedish Museum of Natural History (NRM), University of Kansas Museum of Natural History (Kan, KUNHM), University of Washington, Burke Museum (UWBM), Uppsala University (UU), Western Australian Museum (WAM).

**The paleognathous taxa are assumed to be VS based on available MSP data from ostrich *Strutio camelus* [22] and aa sequence similarity.

## Table references

1 Aidala Z, Huynen L, Brennan PLR, Musser J, Fidler A, Chong N, Machovsky Capuska GE, Anderson MG, Talaba A, Lambert D, Hauber ME: **Ultraviolet visual sensitivity in three avian lineages: paleognaths, parrots, and passerines.** *J Comp Physiol A.* 2012, **198**:495-510.

2 Ödeen A, Håstad O: **Complex distribution of avian color vision systems revealed by sequencing the SWS1 opsin from total DNA**. *Mol Biol Evol* 2003, **20**:855-861.

3 Ödeen A, Hart NS, Håstad O: **Assessing the use of genomic DNA as a predictor of the maximum absorbance wavelength of avian SWS1 opsin visual pigments**. *J Comp Physiol A* 2009, **195**: 167-173.

4 Okano T, Kojima D, Fukada Y, Shichida Y, Yoshizawa T: **Primary structures of chicken cone visual pigments: Vertebrate rhodopsins have evolved out of cone visual pigments.** *Proc Natl Acad Sci USA* 1992*,* **89**:5932-5936.

5 Machovsky Capuska GE, Huynen L, Lambert D, Raubenheimer D: **UVS is rare in seabirds**. *Vision Res* 2011, **51**, 1333-1337.

6 Yokoyama S, Radlwimmer FB, Blow NS: **Ultraviolet pigments in birds evolved from violet pigments by a single amino acid change**. *Proc Natl Acad Sci USA* 2000, **97**, 7366-7371.

7 Håstad O, Ernstdotter E, Ödeen A: **Ultraviolet vision and foraging in dip and plunge diving birds**. *Biol Lett* 2005, **1**:306-309.

8 Ismar SMH, Chong NL, Igic B, Baird K, Ortiz-Catedral L, Fidler AE, Hauber, ME: **Visual sensitivity, coloration and morphology of red-tailed tropicbirds *Phaethon* *rubricauda* breeding on the Kermadec Islands**. *New Zealand J Zool* 2011, **38**:29-42.

9 Wright SG, Dearborn DC: **Male ornament variation in a sexually dimorphic seabird with variable male mating success**. *Evol Ecol* *Res* 2009, **11**:759-770.

10 Ödeen A, Håstad O, Alström P: **Evolution of ultraviolet vision in the largest avian radiation - the passerines**. *BMC Evol Biol* 2011, **11**:313.

11 Ödeen A, Håstad O, Alström P: **Evolution of ultraviolet vision in shorebirds (Charadriiformes)**. *Biol Lett* 2010, **6**:370-374.

12 Håstad O, Partridge JC, Ödeen A: **Ultraviolet photopigment sensitivity and ocular media transmittance in gulls, with an evolutionary perspective**. *J Comp Physiol A* 2009, **195**:585-590.

13 Ödeen A, Håstad O: **Pollinating birds differ in spectral sensitivity**. *J Comp Physiol A* 2010, **196**:91-96.

14 Kawamura S, Blow NS, Yokoyama S: **Genetic analyses of visual pigments of the Pigeon (*Columba livia*)**. *Genetics* 1999, **153**:1839-1850.

15 Carvalho LS, Knott B, Berg ML, Bennett ATD, Hunt DM: **Ultraviolet-sensitive vision in long-lived birds**. *Proc R Soc B* 2011, **278**:107-114.

16 Wilkie SE, Vissers PMAM, Das D, DeGrip WJ, Bowmaker JK, Hunt D: **The molecular basis for UV vision in birds: spectral characteristics, cDNA sequence and retinal localization of the UV-sensitive visual pigment of the budgerigar (*Melopsittacus undulatus*)**. *Biochem. J* 1998, **330**:541-547.

17 Seddon N, Tobias JA, Eaton, M, Ödeen A: **Human vision can provide a valid proxy for avian perception of sexual dichromatism**. *Auk* 2010, **127**:283-292.

18 Ödeen A, Pruett-Jones S, Driskell AC, Armenta JK, Håstad O: **Multiple shifts between violet and ultraviolet vision in a family of passerine birds with associated changes in plumage coloration**. *Proc R Soc B* 2012, **279**:1269-1276.

19 Ödeen A, Håstad O: **New primers for the avian SWS1 pigment opsin gene reveal new amino acid configurations in spectral sensitivity tuning sites**. *J Hered* 2009, **100**:784-789.

20 Das D, Wilkie SE, Hunt, DM, Bowmaker JK: **Visual pigments and oil droplets in the retina of a passerine bird, the canary *Serinus canaria*: microspectrophotometry and opsin sequences**. *Vision Res* 1999, **39:**2801-2815.

21 Wilkie SE, Robinson PR, Cronin TW, Poopalasundaram S, Bowmaker JK, Hunt DM: **Spectral tuning of avian violet- and ultraviolet-sensitive visual pigments**. *Biochemistry* 2000, **39**:7895-7901.

22 Wright M, Bowmaker JK: **Retinal photoreceptors of paleognathous birds: the ostrich (*Strutio camelus*) and rhea (*Rhea americana*)**. *Vision Res* 2001, **41**:1-2.
